# Antimicrobial NIR-Responsive Hydrogels Based on Gellan Gum and Bis-MPA Polyester Dendrimers

**DOI:** 10.1021/acsami.5c02386

**Published:** 2025-04-07

**Authors:** Giuseppina Biscari, Natalia Sanz Del Olmo, Fabio S. Palumbo, Raimondo Gaglio, Giuliana Garofalo, Giovanna Pitarresi, Calogero Fiorica, Michael Malkoch

**Affiliations:** † Department of Biological Chemical and Pharmaceutical Science and Technology (STEBICEF), University of Palermo, Via Archirafi 30-32, Palermo 90123, Italy; ‡ School of Engineering Sciences in Chemistry, Biotechnology and Health (CBH), Department of Fibre and Polymer Technology, Division of Coating Technology, KTH Royal Institute of Technology, Teknikringen 56, Stockholm SE-100 44, Sweden; § Department of Agricultural, Food and Forest Sciences (SAAF), Università degli Studi di Palermo, Viale delle Scienze, Palermo 90128, Italy

**Keywords:** wound healing, gellan gum, antimicrobial dendrimer, hydrogel, antibacterial, radical scavenging

## Abstract

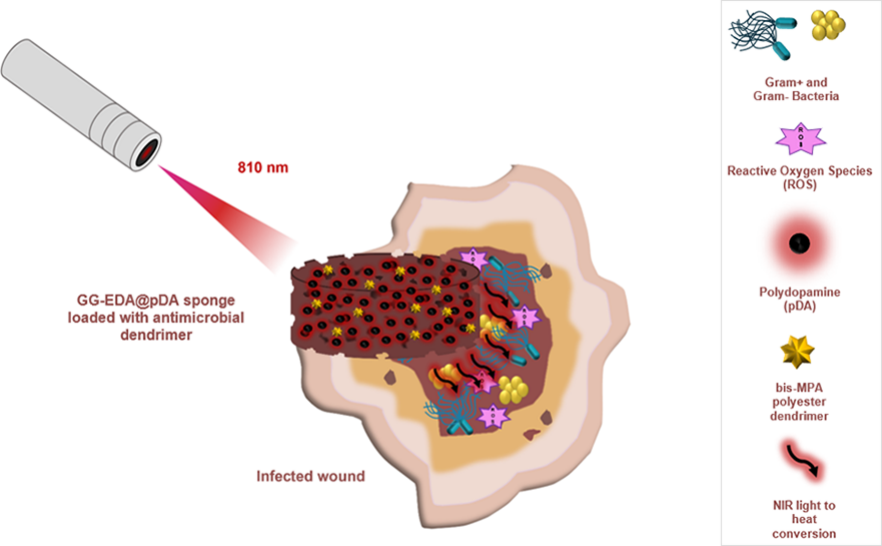

In this study, a near-infrared (NIR)-responsive hydrogel based on ethylenediamine (EDA)-functionalized Gellan Gum was developed through a simple preparation method. This hydrogel incorporates in situ synthesized polydopamine (pDA) and was loaded with first- and second-generation antimicrobial bis-MPA polyester dendrimers (TMP-G1-[Cys]_6_ and MP-G2-[Cys]_12_), bearing cysteamine hydrochloride as peripheral functional groups. The intrinsic ability of pDA to scavenge reactive oxygen species (ROS) and convert NIR light at 810 nm into heat imparted radical scavenging activity and photothermal properties to the systems. It has been demonstrated that, due to the noncovalent interactions with both GG-EDA and pDA, dendrimers are retained differently within the sample depending on their molecular weight and the number of terminal positive charges. This difference in retention influences their antimicrobial activity against *Pseudomonas aeruginosa* and *Staphylococcus aureus*. In particular, it has been shown that the NIR-induced photothermal effect plays a crucial role in triggering the activity of the sample loaded with the most retained dendrimer, which possesses the highest number of terminal positive charges. The high physiological fluid absorption capacity makes these materials ideal for wound exudate management. In addition, their resistance to hydrolytic degradation can be exploited to reduce the frequency of dressing changes, potentially improving patient comfort. The dendrimer-loaded samples demonstrated low cytotoxicity toward human fetal dermal mesenchymal stromal cells (FD-MSCs) and human epidermal keratinocytes (HaCaT). These findings suggest that GG-EDA@pDA+TMP-G1-[Cys]_6_ or TMP-G2-[Cys]_12_ could be promising candidates for the treatment of infected skin wounds.

## 1 Introduction

Skin plays an essential role as a protective barrier against injuries and external aggressions.^1^ In the case of wounds, proper healing is critical to avoid complications such as infection and mortality, which are further exacerbated by the growing challenge of antimicrobial resistance (AMR).^2^ According to the WHO, around 5 million people die each year from unhealed wounds, prompting research into new treatments.^3^ Despite the progress, many studies focus exclusively on the antibacterial effects of cutaneous wound dressing.^4^ Instead, it is essential to develop multifunctional wound dressings that promote complete healing by combining exudate absorption, free radical scavenging,^5^ and effective broad-spectrum antibacterial activity, enhanced through synergistic mechanisms such as photothermal antibacterial action (PTA) and specific intrinsic antimicrobial properties.

Preformed solid dressings, such as gauze and bandages, which are sterilized and ready to use, are preferred in clinical and domestic settings where efficiency and simplicity are essential.^6^ However, these solutions have a limited absorptive capacity.^7^ Although injectable hydrogels offer advantages such as adapting to the shape and depth of the wound (in situ forming hydrogels) and managing larger amounts of exudate, they have the disadvantage of requiring a complex preparation process, which involves mixing components with special syringes and carefully controlling the gelation time and viscosity of the system. Also, the use of injectable hydrogels in some cases could overhydrate the wound bed creating optimal condition for bacterial proliferation.^8^ Dehydrated hydrogels not only help maintain an optimal moisture balance on the wound but also reduce the risk of infection and improve exudate management, promoting safer and more effective healing.^9^ Materials based on polysaccharides derived from plants, algae, or bacterial fermentation are excellent candidates for the development of xerogels, aerogels ,or freeze-dried sponges as wound dressings due to their low cost, wide availability, and versatility.^10^

Gellan Gum (GG) is a well-known anionic exopolysaccharide produced by the fermentation of *Sphingomonas elodea*.^11^ This tetrasaccharide has a linear structure, with its repeating unit consisting of two subunits of β-d-glucose, one subunit each of β-d-glucuronate and α-l-rhamnose.^12^ Due to its biocompatibility, biodegradability, gelation tunability, and mechanical properties, GG is used as a scaffold material in tissue repair, providing a viable alternative for the treatment of skin wounds.^13^ Chemical modifications of GG improve its physical stability, flexibility, and dispersibility in water.^14^ Recently, a low molecular weight GG derivative functionalized with ethylenediamine (GG-EDA) has been developed to obtain hydrogels with better hydrolytic stability and increased elasticity due to the interaction of amine groups with the glucuronic acid moieties of the tetrasaccharide repeating unit.^15^

Polydopamine (pDA)-based nanomaterials are increasingly being investigated for their ease of preparation, low cytotoxicity, high biocompatibility, strong near-infrared (NIR) absorption,^16^ thermal stability,^17^ high photothermal conversion efficiency,^18^ and radical scavenging activity.^19^ In addition to their widespread use in cancer therapy, recent studies are exploring their antibacterial potential using photothermal therapy (PTT).^20^ By absorbing NIR light between 700 and 1400 nm and converting it into heat, these materials damage bacterial membranes, increase permeability, and cause denaturation of proteins and cytoplasmic enzymes, thereby reducing side effects.^21^ Polymers with pendent amine groups can facilitate dopamine self-polymerization reactions by promoting the formation of intermolecular bonds between dopamine monomers.^22^ Harvey et al. synthesized pDA nanoparticles whose formation was stabilized by a poly(ethylene glycol) polymer chain with two terminal primary amine groups, and the polymer and pDA were observed throughout the volume of the nanoparticles as shown by time-of-flight secondary ion mass spectrometry (ToF-SIMS), indicating that composite structures composed of pDA nanodomains could also be formed.^23^

In the search for materials with strong antibacterial potential, antimicrobial peptides,^24^ metallic nanoparticles,^25^ and highly branched dendrimers^26^ have emerged as promising alternatives to the use of common antibiotics. Dendrimers with multiple terminal ammonium groups have demonstrated significant antibacterial properties; these groups are believed to multivalently interact with the negatively charged bacterial membrane, leading to its disruption and ultimately causing bacterial cell death.^27^ Among these, 2,2-bismethylolpropionic acid (bis-MPA) dendrimers with terminal amine groups stand out as particularly effective macromolecules, capable of preventing bacterial infections without the need for antibiotics.^28^ Recently, Namata et al. synthesized a new family of amino-functionalized bis-MPA dendrimers modified with cysteamine hydrochloride, which exhibited high hydrolytic stability and antibacterial activity, proving to be nontoxic at effective concentrations against Gram-positive and Gram-negative bacteria.^29^

Conceptually, the use of bis-MPA dendrimers,^30^ infused as antibacterial agents into polysaccharide dried sponge with the ability to absorb exudate while scavenging free radicals and exerting photothermal antibacterial activity, could offer significant advantages over existing wound healing solutions. This approach would allow for sustained antibacterial action, reducing the need for repeated application of antimicrobial agents to the wound site, and ensure prolonged protection against infections.

In this work, we developed a new material platform, where the matrix consisted of GG-EDA incorporating in situ synthesized pDA and subsequently enriched with first- and second-generation antimicrobial polyester bis-MPA dendrimers (TMP-G1-[Cys]_6_ and TMP-G2-[Cys]_12_) containing 6 and 12 cysteamine hydrochloride as peripheral groups, respectively. The main goal was to develop advanced multifunctional sponges for the treatment of topical skin wounds, with the ability to simultaneously absorb exudate, neutralize ROS, and counteract bacterial infection. Rheological measurements, Fourier-transform infrared (FT-IR) spectroscopy, swelling, and hydrolytic degradation studies were used to characterize the dried sponges and the resulting hydrogels. The photothermal effect resulting from the presence of pDA in the samples was demonstrated by irradiating the hydrogel with an NIR laser. In addition, a colorimetric assay (ABTS) was used to evaluate the radical scavenging ability of the produced material. Microbiological evaluations of the hydrogels were conducted on Gram-negative (*Pseudomonas aeruginosa*) and Gram-positive (*Staphylococcus aureus*) bacterial strains, and cytocompatibility was studied using two different cell lines (FD-MSC and HaCaT).

## 2 Results and Discussion

### 2.1 Production of GG-EDA@pDA Nanocomposite Hydrogel and Derived Sponge

**Figure 1:**
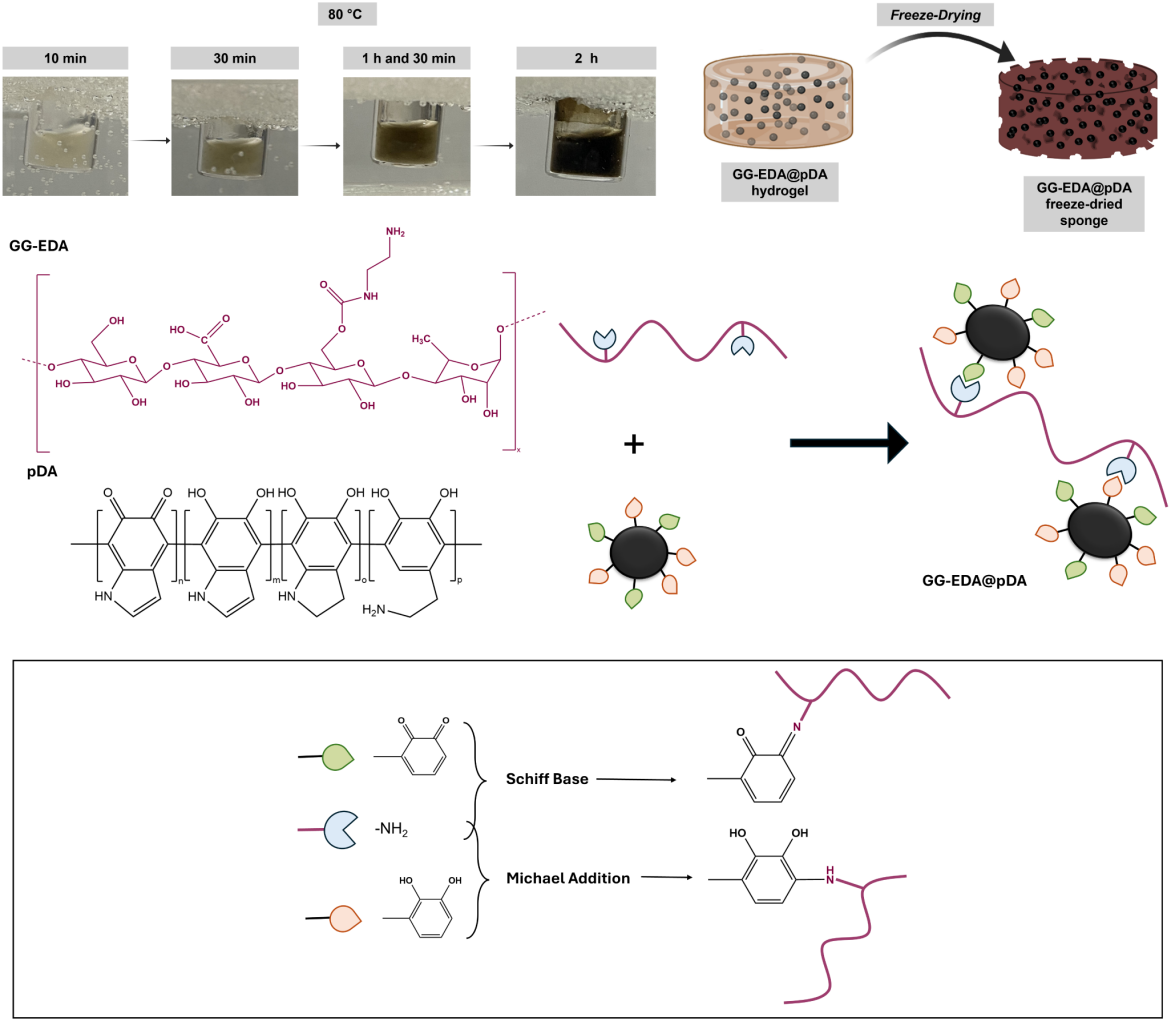
*In situ* production of pDA nanoparticles (top) and possible interaction scheme between pDA and GG-EDA (bottom).

In our previous work, injectable composite hydrogels of low molecular weight GG-EDA and pDA were obtained by dispersing the preformed nanoparticles in the gel-forming solution and utilizing vinyl sulfone-functionalized multi-arm PEG as cross-linker.^31^ To further develop a simpler and more versatile production process, dopamine oxidative polymerization was instead initiated within the GG-EDA *sol* at 80 °C. This approach likely allows the pendant amine groups of GG-EDA to form covalent bonds with the “growing” pDA moieties through Michael-type additions and Shiff base formation (Figure 1), thereby anchoring the photothermal agent to the main hydrated polymeric network and achieving composite hydrogel production via a “one-pot” procedure. The progression of in situ dopamine polymerization was visually evident, as shown in Figure 1. The appearance of the dispersion changes over time, transitioning from a light to a dark brown color within 2 h, indicating the formation of pDA-based nanostructures.^16^ By exploiting the thermotropic properties of GG and the peculiar physicochemical properties of GG-EDA, using a concentrated *sol* (6% w/v), stable and easy-to-handle hydrogels were obtained by cooling the polymeric dispersion to room temperature without the need for an ionotropic curing process, which is typically required for Gellan Gum-based hydrogels. Finally, the freeze-drying procedure makes it possible to obtain dried samples with a uniform microporous structure that imparts sponge-like features, making it especially well-suited for applications such as wound dressing (SEM analysis is shown in Figure S1).

### 2.2 Characterization of GG-EDA@pDA Nanocomposite Freeze-Dried Sponge and Derived Hydrogel

**Figure 2:**
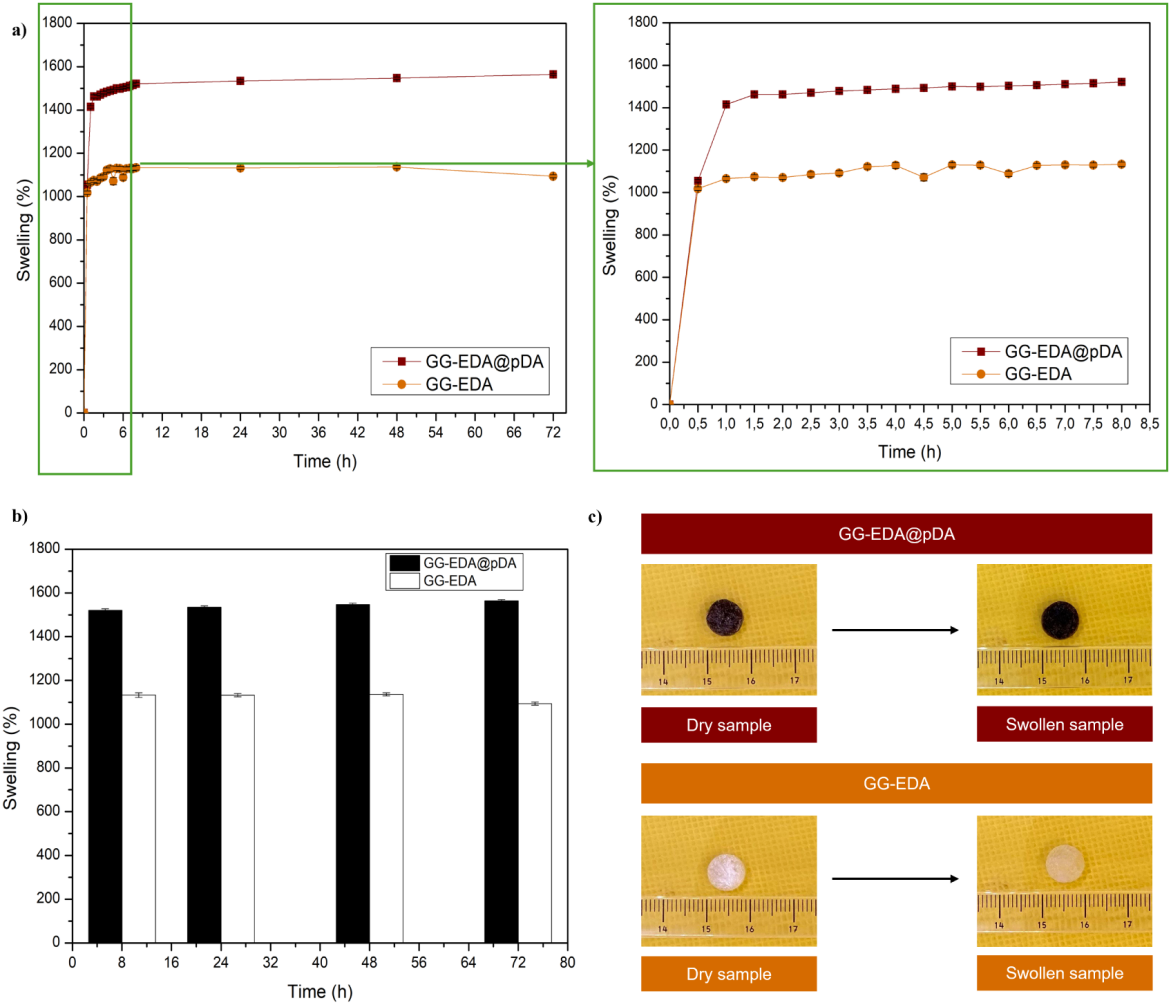
Swelling % (a-, b), photographs of dry and swollen samples (c) of GG-EDA@pDA and GG-EDA. All data are shown as a mean value ± SD (*n* = 3).

The high adsorption capacity of the GG-EDA@pDA sponges was evaluated by a swelling study using GG-EDA as a control (Figure 2a). All samples reached equilibrium swelling within 8 h. GG-EDA@pDA sponges showed an increase of more than 1500%, while GG-EDA reached a maximum swelling of about 1300%. Although the amine groups present in the polysaccharide structure contribute to water retention through hydrogen bonding, the introduction of pDA, rich in hydroxyl, amino, and quinone groups, seems to further promote the formation of molecular interactions that increase the free space in the network, thus increasing the adsorption capacity. As shown in Figure 2b, the systems maintain their stability under physiological conditions. Figure 2c shows pictures of the dry and swollen samples, macroscopically showing that although the system absorbs a large amount of DPBS, it retains its shape. This stability is essential to ensure the effectiveness of the material in wound healing and avoid potential side effects. The balance between liquid absorption and structure maintenance makes it suitable for use in a physiological environment. These results suggest that the obtained sponges could potentially absorb a substantial amount of exudate, thereby reducing the risk of tissue maceration and inhibiting bacterial proliferation.^32^

**Figure 3:**
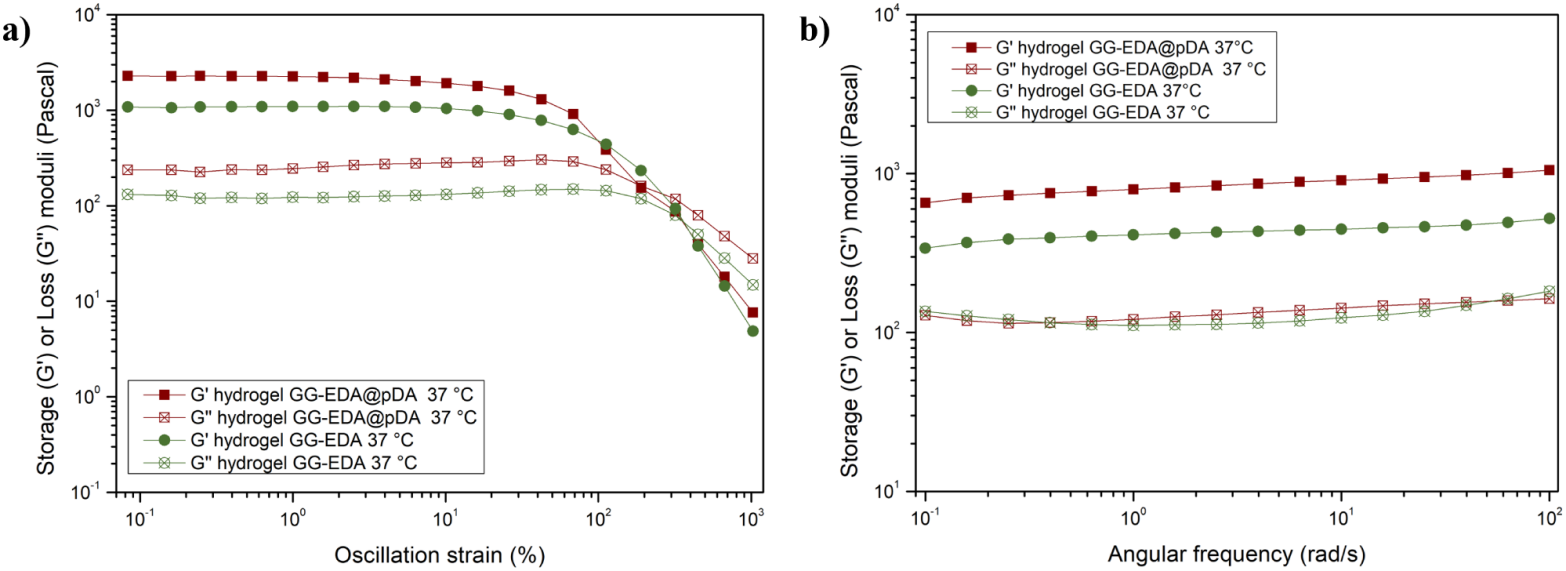
Oscillation amplitude (a) and frequency sweep (b) analyses at 37 °C of GG-EDA and GG-EDA@pDA.

The viscoelastic behavior of hydrogels obtained by swelling the GG-EDA@pDA and GG-EDA (as control) sponges in DPBS (pH 7.4) was analyzed through rheological studies conducted under different conditions. This comparison was carried out to investigate whether the new interactions formed between the polymeric chains during *in situ* dopamine polymerization could influence the viscoelastic properties. All analyses were conducted at both 25 and 37 °C to identify any differences in the behavior of the system at room temperature versus body temperature. Specifically, amplitude oscillation analysis was performed (Figures 3a and S2a) to evaluate the variation of the storage modulus (*G*′) and loss modulus (*G*″) as a function of the strain% applied to the sample. Hydrogels exhibited strain-dependent viscoelastic behavior at both 25 and 37 °C. At low strain % applied to the samples, *G*′ was about 1 order of magnitude higher than *G*″, indicating that the system behaves as a stable hydrogel with predominantly elastic behavior.^33^ By performing the amplitude sweep, the linear viscoelastic critical region (LVR) was determined.^34^ The LVR, along with the *G*’ and *G*″ values of both samples, is comparable. Notably, beyond the crossover point both samples exhibit nonlinear deformation. Specifically, the elastic modulus of each sample decreases once it surpasses the LVR, continuing until it reaches the crossover point, at which the sample yielded. This behavior is characteristic of other Gellan Gum-based systems, as reported in the literature.^35^

The relatively high strain value (>10^2^ %) at the crossover point demonstrates that a significant force is required to irreversibly deform the hydrogels, indicating that the samples can maintain their structural integrity when applied to a wound bed. Interestingly, the initial 6% (w/v) dispersion concentration of the GG-EDA polymer leads to the formation of very compact hydrogels. At these concentrations, the numerous physical interactions between the polymer chains seem to mask the presence of any new interactions resulting from the integration of pDA. In other words, the network of preexisting physical bonds between the polymer chains reduces the perception of any pDA-induced structural effects due to their high density. This phenomenon highlights how, in a concentrated system, the interactions between the polymer chains dominate over the contribution of additional interactions with the pDA, maintaining the structural integrity of the hydrogels. Furthermore, the *in situ* polymerization of DA does not affect the viscoelastic properties of the system. At 25 and 37 °C, the presence of pDA does not significantly alter either the storage modulus or the loss modulus, indicating that the hydrogel structure remains stable. Focusing on the LVR, the difference in behavior with varying frequency was investigated by applying a strain value below the crossover point using frequency sweep analysis (Figures 3b and S2b). This approach allowed for the evaluation of the structural stability of the GG-EDA and GG-EDA@pDA hydrogels. Throughout the entire frequency range tested, it was observed that *G*’ remained higher than *G*″ (*G*’ ∼ 657 Pa and *G*″ ∼ 128 Pa for GG-EDA@pDA; and *G*’ ∼ 340 Pa and *G*″ ∼ 136 Pa for GG-EDA), supporting the hypothesis that once the three-dimensional polymer network is formed, the samples maintain their elastic behavior throughout the frequency range tested.^36^ Thus, the hydrogel structure remains stable, with *G*’ and *G*″ values being similar for both GG-EDA and pDA-containing hydrogels.

**Figure 4:**
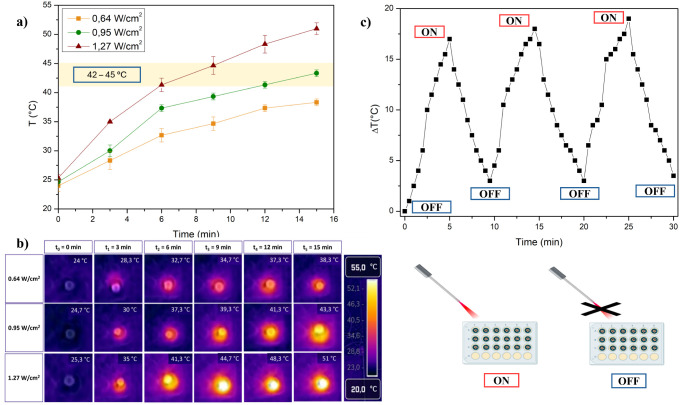
Photothermal effect of GG-EDA@pDA hydrogel, after irradiation at powers of 0.64, 0.95, and 1.27 W/cm^2^ (a); images taken with the FLIR C5 thermal camera (b); photothermal stability studies of the GG-EDA@pDA hydrogel by irradiation-cooling cycles at the power of 1.27 W/cm^2^ (c). All data are shown as a mean value ± SD (*n* = 3).

The photothermal properties of GG-EDA@pDA hydrogels were evaluated using NIR irradiation with an 810 nm laser, monitoring the temperature change of the aqueous medium in contact with the sample over 15 min. The presence of pDA, which absorbs efficiently in the NIR due to its indole-5,6-quinone conjugated structures, makes the GG-EDA@pDA hydrogel photoresponsive and potentially suitable for antibacterial photothermal therapy (PTTA).^37^ Temperatures above 47 °C ensure effective antibacterial activity, while a range of 37–40 °C promotes the expression of heat shock proteins (HSP), accelerating wound healing.^38^ Furthermore, temperatures exceeding 40 °C can enhance the efficacy of antibacterial molecules by increasing the permeability of the bacterial cell wall. As shown in Figure 4a,b, the temperature range of 42–45 °C is reached within 6–8 min at 1.27 W/cm^2^ irradiation power, while lower powers require longer times: 12–15 min at 0.95 W/cm^2^ and more than 15 min at 0.64 W/cm^2^. At 1.27 W/cm^2^, a temperature of over 50 °C is reached after 15 min. These results were confirmed by thermal camera images taken at different irradiation powers.

To evaluate the ability to maintain the hyperthermic effect over time, a photothermal stability study was performed by using consecutive irradiation cycles. A power density of 1.27 W/cm^2^ was applied, with three on–off cycles of 10 min each, consisting of 5 min of irradiation followed by 5 min of cooling. As shown in Figure 4c, the temperature in the well increased and decreased consistently after each cycle. At the end of each irradiation phase, the temperature reached a Δ*T* of approximately 18–20 °C, while after the cooling phase, it returned to a value like the initial temperature (25 °C). GG-EDA@pDA hydrogel could therefore be useful in the treatment of chronic wounds, allowing repeated and on-demand temperature control to enhance the antimicrobial effect of active agents and improve wound healing through repeated irradiation cycles.^17^ Given the thermotropic behavior of GG, a temperature sweep analysis was performed to investigate the potential changes in the viscoelastic properties of the samples with varying temperature. This analysis is particularly significant, as pDA imparts photothermal properties to the samples, and it is crucial to determine whether an increase in temperature would induce a gel–sol transition. Specifically, the behavior of GG-EDA@pDA hydrogels was investigated by increasing the temperature from 5 to 50 °C. The GG-EDA without pDA was used as a control. As shown in Figure S3, *G*’ consistently exceeds *G*″, and only a slight decrease in both *G*’ and *G*″ was observed with increasing temperature. These results highlight the minimal impact of the temperature on the compactness of the hydrogels.

### 2.3 Radical Scavenging Activity

**Figure 5:**
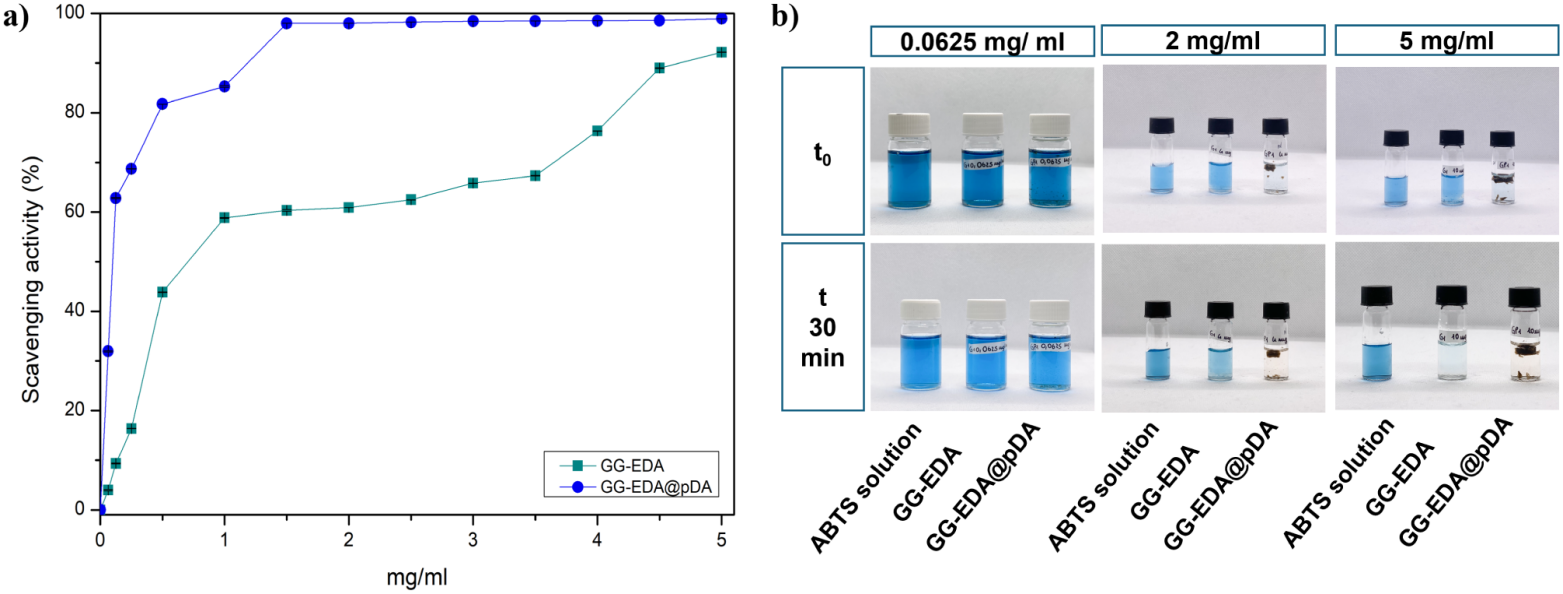
Scavenging activity of GG-EDA@pDA and GG-EDA hydrogels (a); photographs of the analyzed dispersions (b). All data are shown as a mean value ± SD (*n* = 3).

In chronic wounds, a prolonged inflammatory response leads to excessive ROS production by neutrophils and macrophages, delaying healing and re-epithelialization.^39^ Therefore, an effective healing system should have antioxidant properties to limit damage and accelerate tissue repair by blocking oxidation and neutralizing free radicals.^40^ Melanin-inspired materials such as polydopamine (pDA) are effective in scavenging ROS due to the redox reaction of catechol, which eliminates free radicals and forms stable quinone structures.^41^ The antioxidant capacity of GG-EDA@pDA and GG-EDA hydrogels was evaluated using the ABTS assay.^42^ This assay is based on the generation of the ABTS^•+^ cation radical, which appears blue-green and shows an absorbance peak at 734 nm; this radical can be reduced by an antioxidant. When the analyte transfers an electron to the radical, it is reduced to a colorless nonradical molecule. The absorbance of the ABTS^•+^ radical solution is proportional to its concentration.^42^ The recorded absorption spectra show that both GG-EDA and GG-EDA@pDA exhibit dose-dependent antioxidant capacities (Figure S4). The plots show that with increasing concentration, both samples exhibit an increased ability to scavenge radicals (Figure 5a). A clear discoloration of the solutions was observed, indicating the neutralization of ABTS^•+^ radicals (Figure 5b). The antioxidant activity of GG is mainly attributed to its polysaccharide structure.^43^ The incorporation of pDA enhances this effect due to the redox potential of the polycatechol structures on the surface and the temporary formation of internal radicals.^44^ The results indicate that GG-EDA@pDA has a higher radical scavenging activity compared to GG-EDA. Specifically, at a concentration of 1 mg/mL, GG-EDA@pDA scavenges approximately 80% of the radicals, compared to 60% for GG-EDA. At the maximum tested concentration of 5 mg/mL, GG-EDA scavenges 90% of the radicals, while GG-EDA@pDA reaches a plateau of 100% radical scavenging already at 1.5 mg/mL.

### 2.4 Adsorption of TMP-G1-[Cys]_6_ and TMP-G2-[Cys]_12_ Dendrimers Onto GG-EDA@pDA Nanocomposite Freeze-Dried Sponges and Characterization

**Figure 6:**
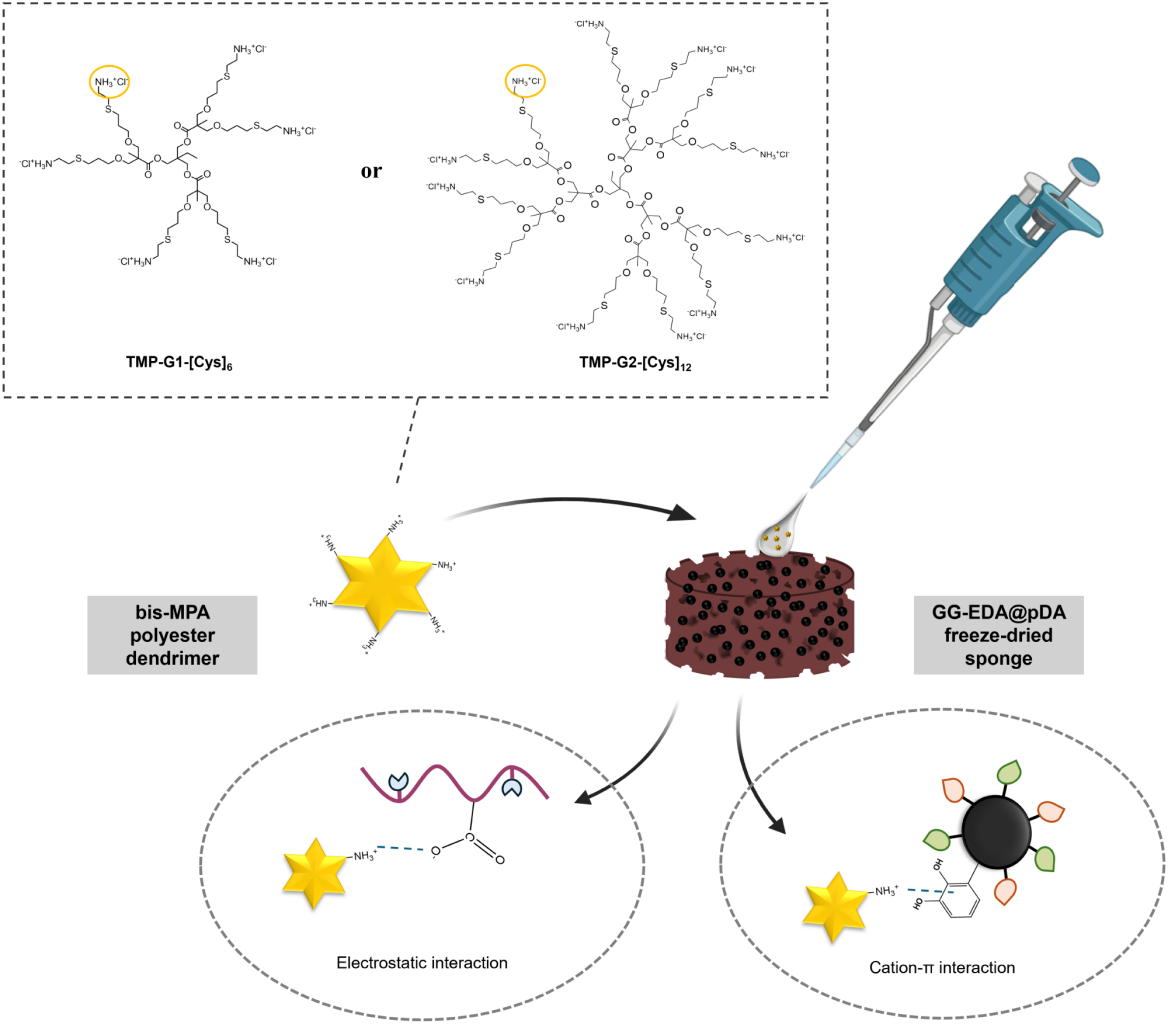
Adsorption of TMP-G1-[Cys]_6_ and of TMP-G2-[Cys]_12_ dendrimers on GG-EDA@pDA freeze-dried sponges.

To enable the hydrogels to exhibit antibacterial activity, antimicrobial dendrimers derived from 2,2-bis(hydroxymethyl)propionic acid (bis-MPA) were adsorbed onto freeze-dried sponges. Specifically, first- and second-generation dendrimers functionalized with cysteamine hydrochloride were employed, featuring 6 (TMP-G1-[Cys]_6_) and 12 (TMP-G2-[Cys]_12_) peripheral amine groups, respectively. These amine groups are protonated at physiological pH, which enhances their antibacterial activity through a mechanism that disrupts bacterial membranes.^45^ TMP-G1-[Cys]_6_ and TMP-G2-[Cys]_12_ were individually adsorbed onto the GG-EDA@pDA sponges by using an adsorption process that exploits the water uptake capacity of the systems. The adsorption of the dendrimers is facilitated by noncovalent physical interactions, such as electrostatic interactions between the protonated amino groups of the dendrimers and the carboxyl groups of GG-EDA, and cation−π interaction between the aromatic groups of polydopamine (which contains phenolic and indole rings, with electron density π) and the protonated amino groups of the dendrimers (Figure 6).^30^ The use of GG as the base polymer provides an optimal support for the adsorption of cationic dendrimers, which interact with the polymer’s carboxylate groups and are integrated into the hydrogel’s three-dimensional structure. This approach allows for the easy preparation of the system before in vivo application by simply rehydrating the freeze-dried sponge with the dendrimer solution. The sponge can be sterilized and stored under sterile conditions in a freezer until needed, ensuring convenience and practicality. This method offers a significant advantage as it does not require specialized expertise from the operator, unlike injectable hydrogels, which often involve complex preparation steps such as mixing different components, adjusting pH, and precise handling factors that increase the risk of user errors.

**Figure 7:**
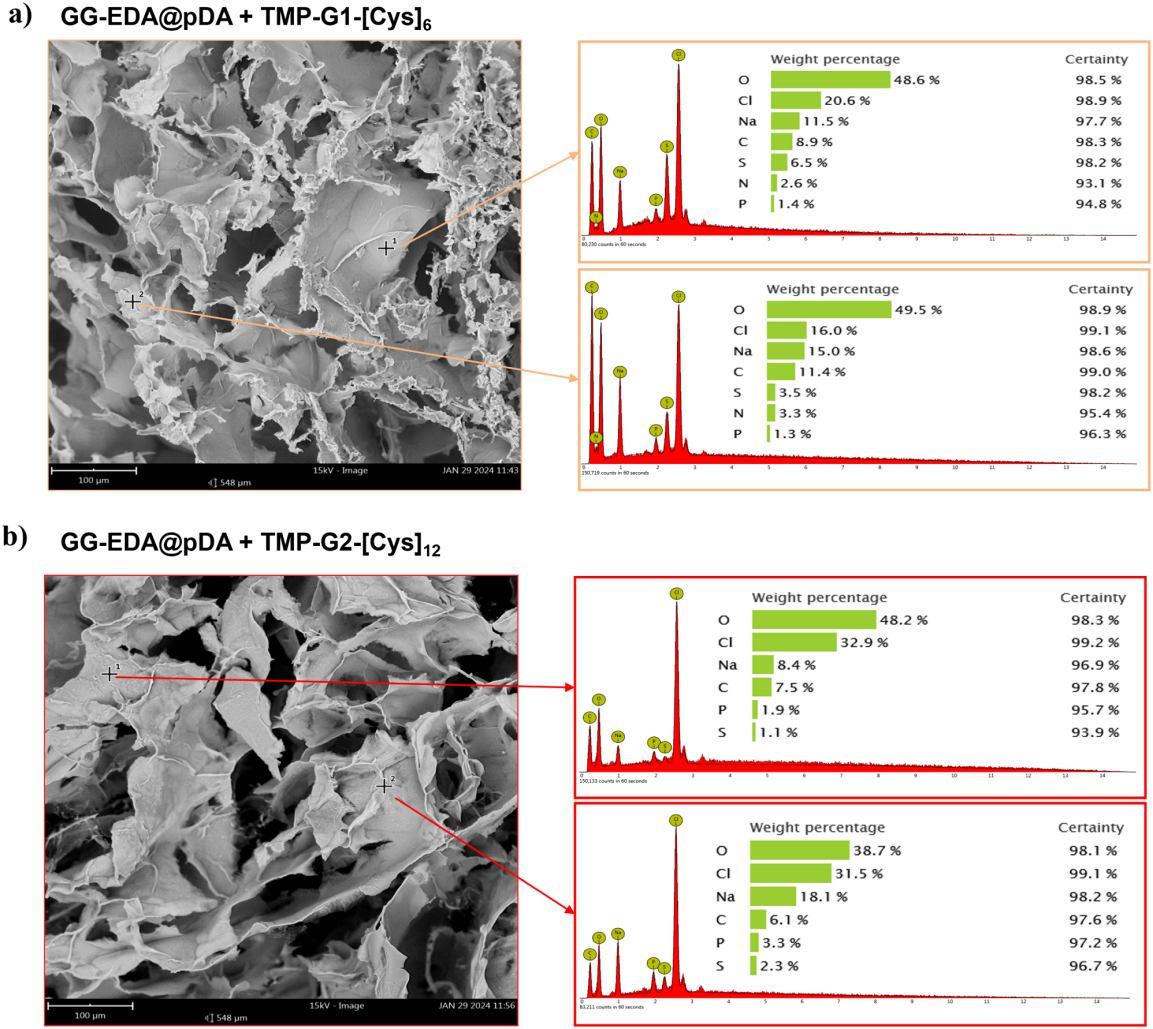
SEM-EDS analysis of GG-EDA@pDA+TMP-G1-[Cys]_6_ (a), and of GG-EDA@pDA+TMP-G2-[Cys]_12_ (b) freeze-dried sponges.

The dendrimers TMP-G1-[Cys]_6_ and TMP-G2-[Cys]_12_ were loaded at 6.5% w/w and 3.25% w/w, respectively,^29^ with respect to the starting polymer. The presence of dendrimers in the GG-EDA@pDA hydrogel was confirmed by ATR-FTIR analysis. Reference samples included the dendrimers TMP-G1-[Cys]_6_, TMP-G2-[Cys]_12_, GG-EDA, and the GG-EDA@pDA. As shown in Figure S5, the band corresponding to the carbonyl stretching vibration (C=O) characteristic of dendrimers, observed at 1727 cm^–1^ and 1743 cm^–1^, is evident in the spectra of both dendrimer-loaded samples. In contrast, this band is absent in the spectra of the GG-EDA and GG-EDA@pDA reference samples. SEM analysis revealed a porous structure in the freeze-dried samples (Figure 7). EDS spectroscopy, performed on both surfaces and at several spots of the samples, confirmed the presence of adsorbed dendrimers. In particular, sulfur, detected in the GG-EDA@pDA+TMP-G1-[Cys]_6_ and GG-EDA@pDA+TMP-G2-[Cys]_12_ samples, is attributed to the dendrimer structures containing cysteamine (for the sake of simplicity, only two spots for each sample are reported in Figure 7a,b).

**Figure 8:**
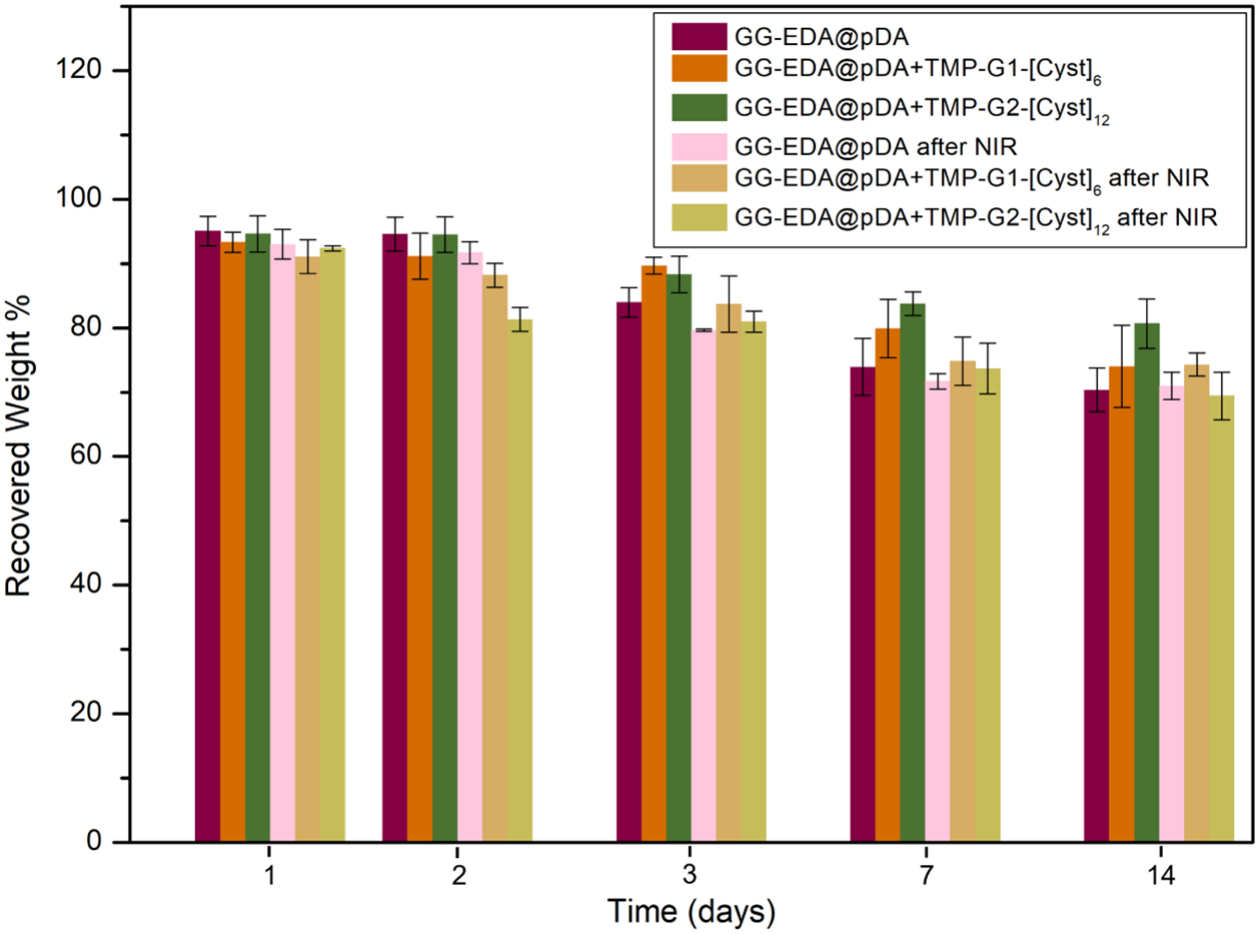
Hydrolytic degradation of GG-EDA@pDA, GG-EDA@pDA+TMP-G1-[Cys]_6_, and GG-EDA@pDA+TMP-G2-[Cys]_12_ hydrogels. All data are shown as a mean value ± SD (*n* = 3).

In general, wound dressings are changed every 7–14 days. However, if the hydrogel is resistant to rapid hydrolytic degradation, the frequency of dressing changes can be reduced, thereby alleviating patient discomfort and pain.^46^ To assess the hydrolytic degradation resistance of the produced systems, hydrogels derived from the hydration of GG-EDA@pDA, GG-EDA@pDA+TMP-G1-[Cys]_6_, and GG-EDA@pDA+TMP-G2-[Cys]_12_ sponges were incubated in DPBS (pH 7.4). Their weight loss was monitored at different time intervals in response to NIR laser irradiation. An increase in the temperature, even for a short time, could potentially accelerate hydrolytic and erosive processes in the hydrogel. Figure 8 shows that the samples exhibited good stability in phosphate buffer with only a 20–30% loss of their initial weight after 14 days. It is evident that hyperthermic effects do not significantly affect the weight loss of the samples.

**Figure 9:**
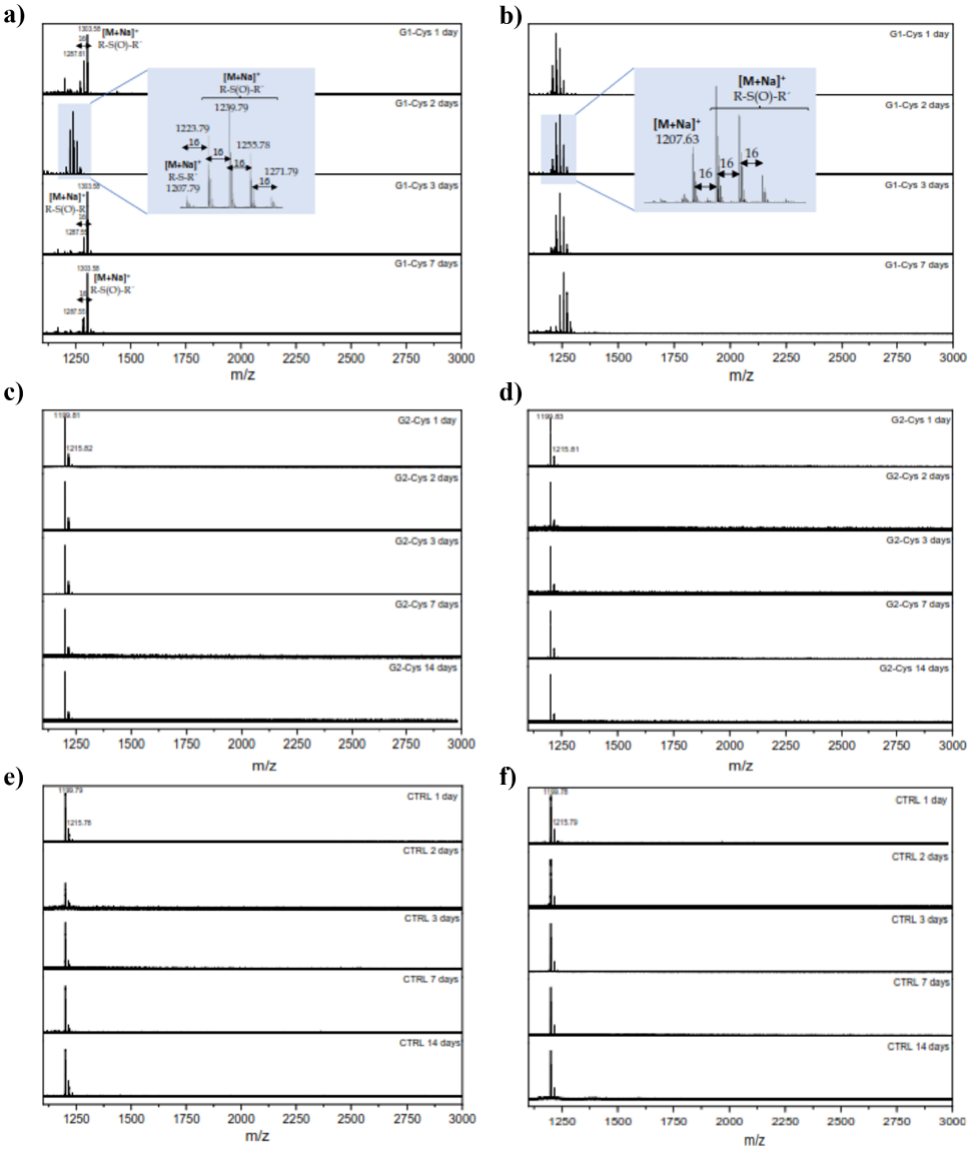
Leach-out analysis of TMP-G1-[Cys]_6_ from GG-EDA@pDA+TMP-G1-[Cys]_6_ hydrogel after NIR treatment (a) and without radiation (b); of TMP-G2-[Cys]_12_ from GG-EDA@pDA+TMP-G2-[Cys]_12_ hydrogel after NIR treatment (c) and without radiation (d) in comparison with control sample (GG-EDA@pDA without dendrimers) after NIR treatment (e) and without radiation (f).

A leaching study of dendrimers from the hydrogels was conducted in DPBS (pH 7.4) and 37 °C. Hydrogels containing first- and second-generation dendrimers, as well as control samples consisting of leachate from the sample without dendrimers, were evaluated after NIR irradiation treatment and without irradiation. However, MALDI confirmed the presence of the TMP-G1-[Cys]_6_ dendrimer in the leachate after 1 day, regardless of whether the sample was irradiated or not (Figure 9a,b). Interestingly, for these samples containing the first-generation dendrimer, MALDI showed a pattern of several peaks with a distance of 16 Da between them. As reported in the literature, thioethers can be oxidized in MALDI to sulfoxides or sulfones.^47^ The molecular peak of the first-generation dendrimer corresponds to [M + Na]^+^ = 1207 Da and all peaks at higher mass can be attributed to oxidized species. In contrast, for the leachate from the hydrogels containing the second-generation derivative (TMP-G2-[Cys]_12_), MALDI did not show any peaks attributed to the dendrimer at any of the selected times or conditions (Figure 9c,d). This result may indicate that the dendrimer remains encapsulated within the hydrogel structure and is not leached out. This could be explained by the larger size of the second-generation dendrimer compared to the first-generation one as well as the higher number of terminal amino groups, which would cause the dendrimer to remain tightly bound to the polymer network. As expected, the control samples did not show any peaks attributed to the presence of bis-MPA dendrimers (Figure 9e,f).

### 2.5 Cytocompatibility and Hemocompatibility of GG-EDA@pDA Nanocomposite Hydrogels Loaded with Dendrimers

**Figure 10:**
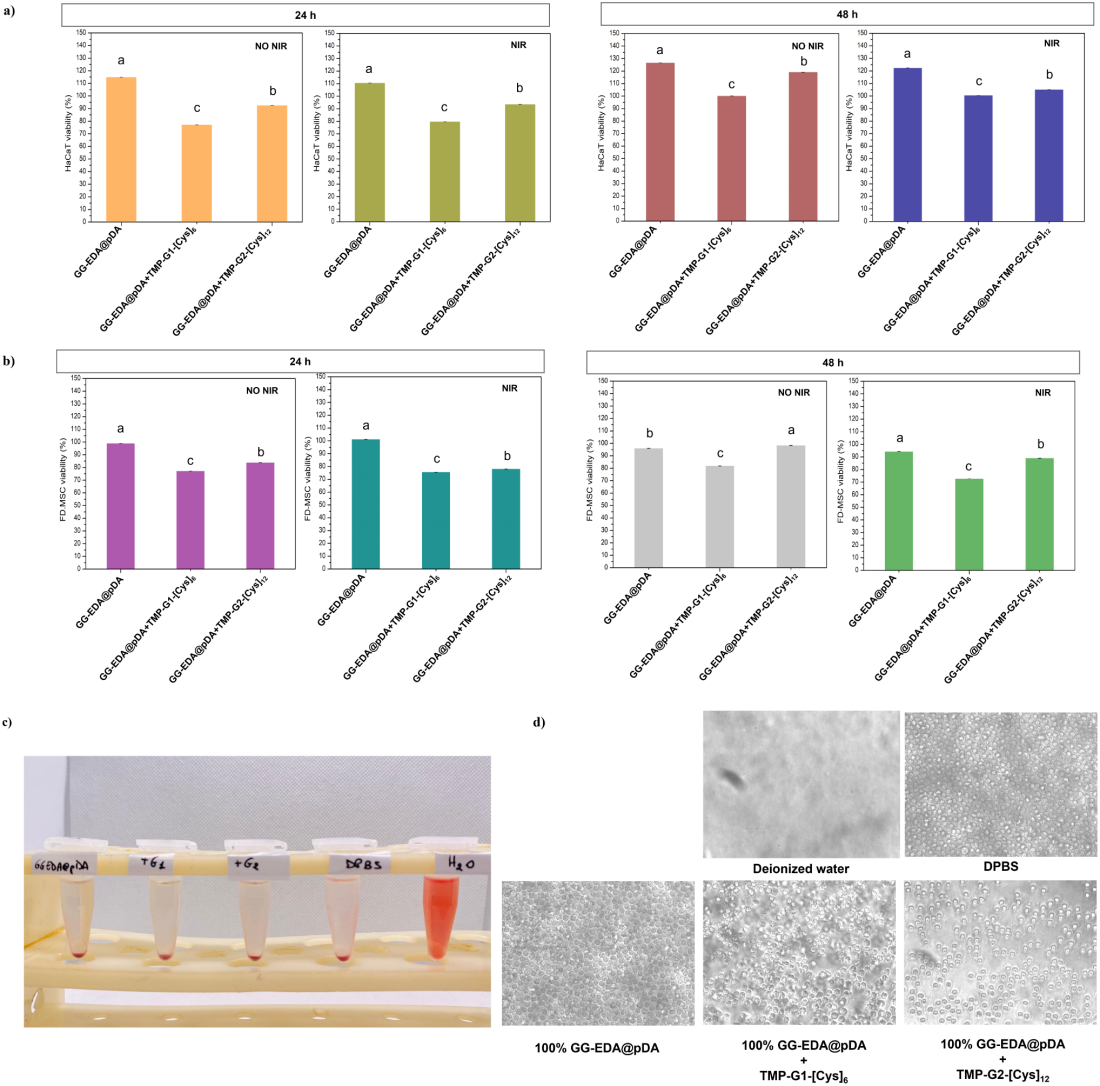
Cytocompatibility evaluation of GG-EDA@pDA, GG-EDA@pDA+TMP-G1-[Cys]_6_, and GG-EDA@pDA+TMP-G2-[Cys]_12_ with HaCaT cells (a) and FD-MSC cells (b) after NIR (8 min at 1.27 W/cm^–1^) and non-NIR irradiation, after 24 and 48 h. All data are shown as a mean value ± SD (*n* = 3); different superscript letters indicate significant differences on cell viability according to Tukey’s test between samples for *p* ≤ 0.001. Hemolysis images (c) and red blood cell morphologies (d).

To evaluate and confirm the potential of obtained hydrogels as systems for skin wound application, the cytocompatibility of the samples with human fetal dermal mesenchymal stromal cells (FD-MSCs) and human epidermal keratinocytes (HaCaT) was investigated. Cells were placed in indirect contact for 24 and 48 h, with the GG-EDA@pDA hydrogels and with the dendrimer-loaded hydrogels, GG-EDA@pDA+TMP-G1-[Cys]_6_ and GG-EDA@pDA+TMP-G2-[Cys]_12_. The difference between irradiated (8 min at 1.27 W/cm^–1^) and non-irradiated samples was investigated. Dendrimer-free samples exhibited good biocompatibility with HaCaT and FD-MSC cells after 24 and 48 h of incubation, regardless of NIR irradiation. In the presence of GG-EDA@pDA+TMP-G1-[Cys]_6_, HaCaT cell viability after 24 h was 77% without NIR irradiation and 80% following NIR exposure (Figure 10a). This decrease in viability is likely due to the release of part of the dendrimer payload from the sample into the culture medium, causing low cytotoxic effect. These results indicate that the hydrogels exhibit low cytotoxicity, remaining within the acceptable cytotoxicity limits.^48^ Nonetheless, this effect seems to be temporary, as an increase in cell viability (reaching 100% compared to the control) is observed in both tested conditions after 48 h of incubation. For HaCaT treated with GG-EDA@pDA+TMP-G2-[Cys]_12_, viability values after 24 h were higher compared to those observed with the first-generation dendrimer, reaching 93% without NIR irradiation and 119% with NIR treatment. After 48 h, the viability values were more comparable, at 94% (without NIR) and 105% (with NIR). This result also seems to be consistent with the data obtained from the leach-out analysis (Figure 9), which shows that there is essentially no release of the dendrimer from the sponge. The results obtained for FD-MSC cells (Figure 10b) displayed a similar trend compared with the HaCaT cells. In fact, when cultured in the presence of GG-EDA@pDA+TMP-G1-[Cys]_6_, their viability after 24 h was 77% without irradiation and 76% following NIR exposure. After 48 h, viability levels shifted to 82% and 73%, respectively. Regarding the cells incubated with the second-generation dendrimer, GG-EDA@pDA+TMP-G2-[Cys]_12_, viability was recorded at 84% after 24 h without NIR treatment and 78% following irradiation. Notably, a slight increase in viability was observed after 48 h, with viability values rising to 98% (without NIR) and 89% (with NIR treatment). Through the live/dead staining assay, it was possible to qualitatively observe the presence of viable (stained green) and dead (stained red) cells using different fluorescent probes. It is evident that HaCaT cells retain their polygonal, flat, and enlarged shape with defined edges (Figure S6) and that FD-MSC cells retain their typical fusiform shape (Figure S7).

Fresh venous blood was co-cultured with the hydrogel extract (prepared as described in Section 4.15) to assess blood compatibility. The hemolysis ratios represent the amount of red blood cells that are broken down upon contact with the extract. The data reported in Table 1 indicate that the hydrogel extracts are not hemolytic.^49^ For the positive control (deionized water), the supernatant after centrifugation is red, as shown in Figure 10c, due to the diffusion of hemoglobin from the broken red blood cells into the water, which is also confirmed by the images acquired under the optical microscope, where the absence of erythrocytes is noted (Figure 10d). In the negative control group (DPBS) and in the hydrogel extract group, the supernatants are clear and transparent. The cell morphologies are intact in both the negative control images and the other samples. These results indicate that the hydrogel extracts have good compatibility with blood.

**Table 1:** Hemolytic Ratio of Sample Extracts

Extract sample	Hemolytic ratio of GG-EDA@pDA	Hemolytic ratio of GG-EDA@pDA + TMP-G1-[Cys]_6_	Hemolytic ratio of GG-EDA@pDA + TMP-G2-[Cys]_12_
25%	1.9% ± 0.06	2.7% ± 0.06	1.8% ± 0.05
50%	2.9% ± 0.02	3.4% ± 0.03	3.2% ± 0.03
100%	3.2% ± 0.04	4.4% ± 0.02	3.8% ± 0.05

### 2.6 In Vitro Evaluation of Antibacterial Activity of GG-EDA@pDA Nanocomposite Hydrogels Loaded with Dendrimers

**Figure 11:**
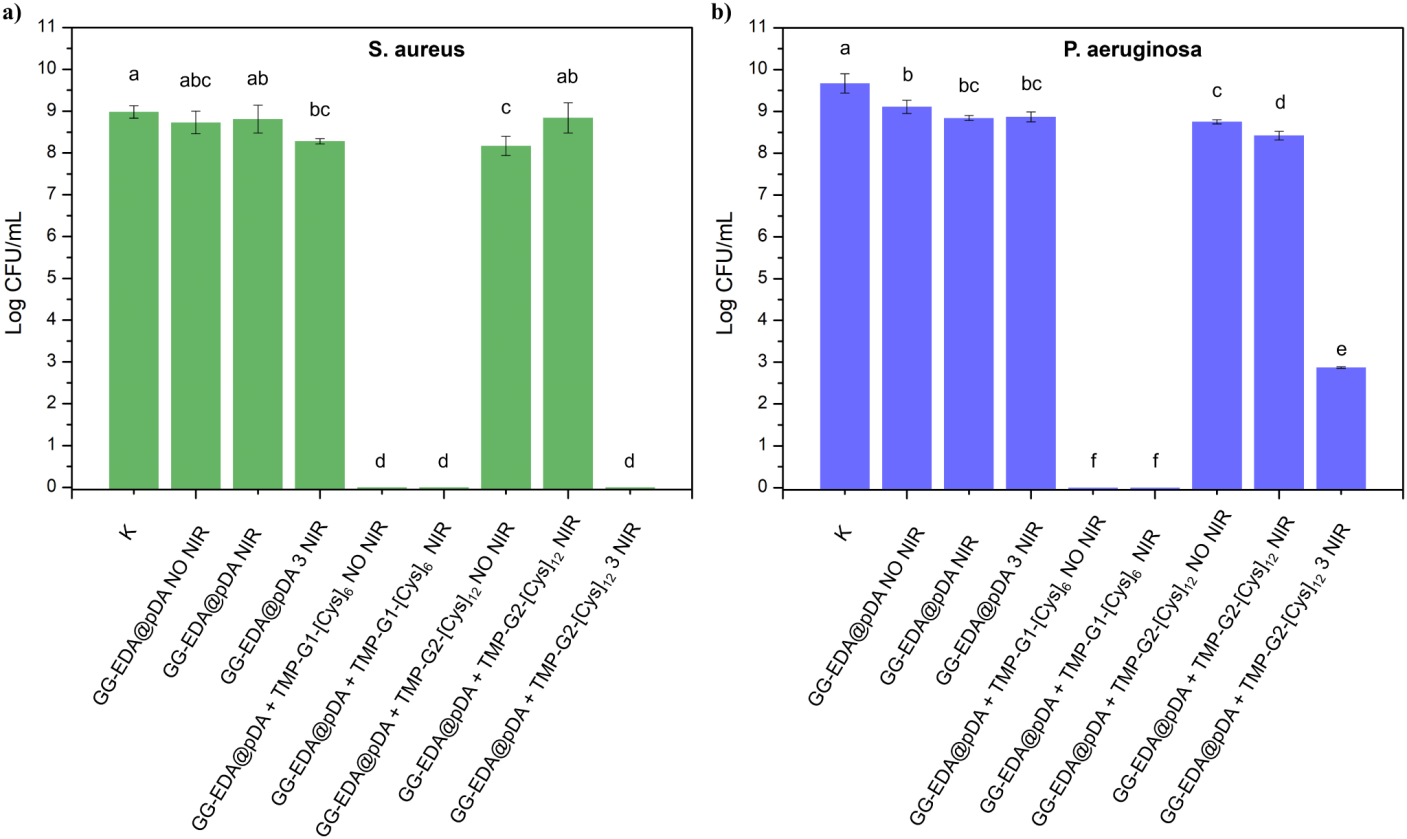
Antibacterial activity against *S. aureus* (a) and *P. aeruginosa* (b) of GG-EDA@pDA, GG-EDA@pDA+TMP-G1-[Cys]_6_, GG-EDA@pDA+TMP-G2-[Cys]_12_ hydrogels, without and after NIR irradiation (1.27 W/cm^2^, 8 min). All data are shown as a mean value ± SD (*n* = 3); different superscript letters indicate significant differences on microbial concentrations according to Tukey’s test between samples for *p* ≤ 0.001.

The inhibitory properties of the hydrogels developed in this study were evaluated *in vitro* against two pathogenic bacterial species, including *S. aureus* among Gram-positive bacteria and *P. aeruginosa* among Gram-negative bacteria. These bacteria represent the main pathogenic agents responsible for the majority of infections associated with skin wounds.^50^ The approach used to evaluate the antibacterial activity of the samples included the application of the plate count method. As shown in Figure 11a,b, all GG-EDA@pDA and GG-EDA@pDA+TMP-G2-[Cys]_12_ whether untreated and irradiated with NIR light for a single treatment did not reveal any antibacterial activity against the two pathogenic bacteria used as indicator microorganisms. However, the hydrogels containing GG-EDA@pDA+TMP-G2-[Cys]_12_ after three NIR treatments reduced at undetectable levels (<1 Log CFU/mL) with the concentration of *S. aureus* and of 6.0 Log cycles of *P. aeruginosa*. The levels of both pathogenic bacteria completely disappeared in samples containing GG-EDA@pDA+TMP-G1-[Cys]_6_, even without irradiation. This finding may be because the dendrimer TMP-G1-[Cys]_6_ is released from the hydrogels in the form of degradation fragments that are still active against bacteria, as evidenced by the leach-out study (Figure 9). In contrast, the dendrimer TMP-G2-[Cys]_12_, due to the higher number of terminal amine groups, remains firmly bound to the polymer network and interacts more intensely both with the polydopamine and the cross-linked polysaccharide network. It is likely to suppose that the decrease in bacterial viability after consecutive NIR treatment is probably due to the loosening of the mesh of the hydrogels, allowing a small amount of dendrimer fragments to be released in the medium. Overall, these results confirm that these materials are highly promising for the treatment of skin wounds at different healing stages. For example, in the initial phases, the hydrogel containing TMP-G1-[Cys]_6_ could be applied, followed by the TMP-G2-[Cys]_12_-loaded hydrogel, which can be activated on demand via near-infrared (NIR) stimulation to further enhance its therapeutic effects.

## 3 Conclusions

In conclusion, nanostructured NIR-responsive hydrogels based on GG-EDA with in situ synthesized pDA nanoparticles have been developed. All of the tested samples exhibited excellent water uptake and hydrolytic resistance for up to 14 days, even under daily NIR irradiation. Rheological studies revealed the viscoelastic behavior of hydrogels, good structural resistance to frequency changes, and a stable elastic modulus up to 50 °C. The presence of pDA confers a photothermal effect. Upon NIR irradiation, samples achieved effective temperatures for potential bacterial inhibition (45 °C in 8 min and 51 °C in 15 min at 1.27 W/cm^2^ and can withstand on–off irradiation cycles without loss of efficiency. The antioxidant properties of the GG-EDA@pDA hydrogels were confirmed by an ABTS assay, showing high radical scavenging capabilities. To endow the hydrogels with antibacterial activity, the TMP-G1-[Cys]_6_ and TMP-G2-[Cys]_12_ dendrimers were adsorbed, which showed good hemocompatibility and low cytotoxity with HaCaT and FD-MSC cells. These systems showed significant antibacterial activity due to the presence of dendrimers and further enhanced by pDA, which can generate a nonspecific photothermal effect after NIR irradiation. Based on these results, we believe that these NIR-responsive nanostructured hydrogels containing dendrimers represent promising materials as advanced dressings for the treatment of skin wounds.

## 4 Experimental Section

### 4.1 Chemicals

Gellan Gum (Gelzan CM), sodium hydroxide (NaOH), Dowex 50WX8 resin, tetrabutylammonium hydroxide (TBA–OH), anhydrous dimethyl sulfoxide (DMSOa), bis(4-nitrophenyl) carbonate (4-NPBC), ethylenediamine (EDA), dopamine hydrochloride (DA), acetone, 2,4,6-trinitrobenzenesulfonic acid solution (TNBS), hydrochloric acid (HCl), deuterium oxide (D_2_O), phosphate buffer (Dulbecco’s Phosphate-Buffered Saline, DPBS), 2,2′-azino-bis(3-ethylbenzothiazoline-6-sulfonic acid) (ABTS), Live/Dead Cell Double Staining Kit, and CellTiter 96 AQueous One Solution Cell Proliferation Assay (MTS) were all purchased from Sigma-Aldrich (Italy).

### 4.2 Bacterial Strains and Cell Lines

To evaluate the antibacterial properties of hydrogels, two strains belonging to the American Type Culture Collection (ATCC) were used as indicator bacteria (sensitive to antimicrobial compounds). Gram-positive (*S. aureus* ATCC33862) and Gram-negative (*P. aeruginosa* ATCC25688) bacteria were chosen. *S. aureus* was propagated in Brain Heart Infusion (BHI) broth incubated at 37 °C for 24 h, while *P. aeruginosa* was propagated in Nutrient Broth (NB) incubated at 25 °C for 24 h. Both media were purchased from Oxoid (Basingstoke, England).

Human fetal dermal mesenchymal stromal cells (FD-MSCs) were isolated from fetal dermis in accordance with a protocol approved by ISMETT’s Institutional Research Review Board (IRRB) and ethics committee (IRRB/00/15), using nonenzymatic tissue outgrowth technique, as described by Chinnici et al.^51^ Human keratinocyte (HaCaT) cells were purchased from Istituto Zooprofilattico Sperimentale della Lombardia e dell’Emilia-Romagna.

### 4.3 Apparatus

Freeze-drying was done using a CoolSafe Pro 95-15 Freeze-Dryer, LaboGene Scandinavian by Design. The proton nuclear magnetic resonance (^1^H NMR) spectra were recorded using a Bruker Avance II 300 instrument (300.12 MHz). FT-IR analyses were performed by using a Bruker Alpha apparatus. UV measurements were carried out using a Shimadzu UV-2401PC spectrophotometer. Rheological analyses were conducted with a DHR2 TA Instruments Trios rheometer using a parallel plate geometry with 20 mm diameter serrated plates and a self-heating Peltier plate. Optical microscope images were acquired using an AxioVert200 microscope (Zeiss). Scanning electron microscopy (SEM) images were acquired with a Phenom XL instrument, Alfatest. Hyperthermic studies were conducted using the Medical Diode Laser System GBox-15A/B. The temperature increase in the irradiated well was measured with a fiber optic temperature probe, CEM Discover SP (±0.2 °C). Thermal images were acquired by using a FLIR C5 thermal camera.

### 4.4 Synthesis of GG-EDA

Low molecular weight GG was produced from high molecular weight Gellan Gum (Gelzan CM) under alkaline conditions for 8 h, as previously reported.^52^ Tetrabutyl ammonium salt (GG-TBA) was prepared as previously reported to aid its dispersion in organic solvents.^53^ The functionalization procedure is similar to the one already described and involves the activation of the primary hydroxyl groups of the glucosidic residues of GG with 4-NBPC and the subsequent reaction with EDA.^53^ The degree of molar derivatization in ethylenediamine (DD_EDA_ mol %) was evaluated by ^1^H NMR analysis and by the TNBS colorimetric assay of GG-EDA. For the H^1^ NMR analysis, GG-EDA was dispersed at 90 °C for 2 min in D_2_O at a concentration of 2% w/v. The degree of molar functionalization in EDA was determined based on the ratio:
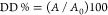
where *A* is the area of the integrals at δ 2.6 ppm and *A*_0_ is the integral of the peak at δ 1.2 ppm, which represents the protons of the methyl group of the rhamnose of GG. The TNBS colorimetric assay was performed according to the supplier’s instructions. GG-EDA was dissolved in Milli-Q water at a concentration of 0.25% w/v. The analyte solution (100 μL) was mixed with TNBS (1 mL) reagent solution, prepared by diluting the supplier’s standard solution 50-fold with borate buffer (0.1 M, pH 9.3). The resulting solution was incubated for 2 h at 37 °C. Subsequently, the absorbance was measured at 500 nm. The analyses were performed in triplicate. A calibration curve was obtained by measuring the absorbance at 500 nm of bisPEG-NH_2_ aqueous solutions at known concentrations, incubated with the TNBS following the procedure just described for the GG-EDA. By correlating the absorbance at 500 nm of the GG-EDA dispersions with the calibration curve from the standard solutions, the degree of functionalization in the EDA groups of the derivative was determined. The test was also performed on GG as a negative control. The ^1^H NMR spectrum of GG-EDA (Figure S8) shows a peak at δ 2.6 ppm, assigned to the protons of the methylene vicinal to the free amino group of EDA. The degree of derivatization (DD_EDA_ mol %) was found to be 28 ± 4 mol %. This value was confirmed by a TNBS colorimetric assay. This reagent reacts with primary amino groups at basic pH to form a product that absorbs at 346 nm. The absorbance is therefore proportional to the number of amino groups present in the sample and allows to determine the percentage of functionalization in EDA of GG-EDA.^54^ As shown in Table S1, the DD_EDA_ mol % values obtained from the two analysis methods are nearly identical.

### 4.5 Production of GG-EDA@pDA Nanocomposite Hydrogel and Derived Sponge

The precursor hydrogels were prepared by dispersing GG-EDA in Milli-Q water to a concentration of 6% w/v in an oven at 90 °C. Then, maintaining a temperature of 80 °C in a water bath with continuous magnetic stirring, a dopamine hydrochloride solution was added to achieve a final concentration of 1% w/v. The pH of the mixture was then adjusted to 8 by using 1 M NaOH to promote dopamine polymerization. The mixture was kept in a water bath with stirring for 2 h. After being cooled, the resulting hydrogels were frozen at −80 °C and freeze-dried, resulting in GG-EDA@pDA sponges. To remove unreacted dopamine, dried hydrogels were washed five times in an orbital shaker at 37 °C for 10 min each, performing first three washes with DPBS (2 mL, pH 7.4) and then after two washes with Milli-Q water (2 mL). Finally, the hydrogels were freeze-dried again. Control hydrogels of GG-EDA alone were also prepared by dispersing the polymer in Milli-Q water to a final concentration of 6% w/v, heating it in an oven at 90 °C, and then cooling it to room temperature. The hydrogels were frozen at −80 °C and freeze-dried to obtain the control sponges. UV–vis spectra recorded for the washing solutions showed a decreasing absorbance at the dopamine-specific peak (λ = 280 nm), which became undetectable after the final wash (Figure S9).^55^ Following the washes, the hydrogels were frozen again at −80 °C and freeze-dried.

### 4.6 Swelling Studies

For the studies of swelling, GG-EDA and GG-EDA@pDA sponges (prepared from 200 μL of precursor hydrogel) were carefully weighed and incubated in DPBS (2 mL, pH 7.4) at 37 °C until 72 h. After each time point, the swollen samples were weighed after the excess of buffer was removed with blotting paper. The swelling percentage (Sw%) was calculated as
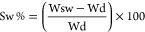


where Wsw is the weight of the sample after swelling and Wd is the weight of the dry sample. Each experiment was performed in triplicate, and the results were expressed as mean value ± standard deviation.

### 4.7 Rheological Characterization

For rheological experiments, the GG-EDA@pDA hydrogels were compared with GG-EDA ones. The swollen samples (after being immersed in 2 mL of DPBS at pH 7.4 for 24 h and dried with filter paper) were placed on a parallel plate geometry with a top plate of 20 mm diameter. The LVR (linear viscoelastic region) was preliminarily assessed by strain sweep experiments at 25 and 37 °C, applying a constant frequency of 1 rad/s and an oscillation strain% between 0.01% and 1000%. The viscoelastic properties were also investigated with a frequency sweep study at 25 and 37 °C in the range of 0.1–100 rad/s applying a constant strain of 10%. The temperature ramp study was carried out in the temperature range from 0 to 50 °C by applying an angular frequency of 1 rad/s and a strain% of 10%. The measurement gap was set at 300 μm for all analyses. All studies were performed in triplicate.

### 4.8 Photothermal Studies

To study the photothermal properties, DPBS (2 mL, pH 7.4) was added to each GG-EDA@pDA hydrogel (prepared in the same way as used for rheological analyses). The samples were irradiated with NIR light at λ = 810 nm using three different irradiation powers (0.64 W/cm,^2^ 0.95 W/cm^2^, and 1.27 W/cm^2^ for 15 min). Similarly, the photothermal stability test was conducted through 10 min (on/off) irradiation cycles (5 min irradiation, 5 min cooling) at a radiation power of 1.27 W/cm^2^. After each irradiation period, temperature changes were measured with a fiber optic temperature probe (CEM Discover SP, ±0.2 °C), and images were captured by using a FLIR C5 thermal camera.

### 4.9 Radical Scavenging Assay

To study the radical scavenging activity of the hydrogels, the ABTS (2,2′-azino-bis(3-ethylbenzothiazolino-6-sulfonic acid)) assay was carried out, following the method already reported.^56^ The ABTS^•+^ radical was generated through an oxidation reaction carried out at room temperature in the dark for 16 h, using equal volumes of ABTS solution (7 mM) and potassium persulfate solution (2.45 mM). This solution was then diluted with Milli-Q water to obtain an ABTS^•+^ radical solution with an absorbance (Abs) of 0.70 ± 0.01 at 734 nm. The study involved placing different quantities of freeze-dried sponges, GG-EDA (as a control), and GG-EDA@pDA in the ABTS^•+^ solution and examining the radical scavenger effect as a function of concentration. After 30 min of incubation in the dark and at room temperature, the absorbance of the solutions at 734 nm was measured. The results were shown in terms of percentage of radical scavenging activity, calculated following the equation:
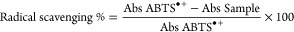


where Abs ABTS^•+^ is the absorbance corresponding to the ABTS^•+^ solution and Abs Sample is the one corresponding to the ABTS^•+^ solution after reacting with the sample.

### 4.10 Adsorption of TMP-G1-[Cys]_6_ and TMP-G2-[Cys]_12_ Dendrimers

Dendrimers (TMP-G1-[Cys]_6_ and TMP-G2-[Cys]_12_) synthesized as described in previous work^29^ were loaded into GG-EDA@pDA sponges by an adsorption method. Aqueous solutions of TMP-G1-[Cys]_6_ (390 μg/100 μL) and TMP-G2-[Cys]_12_ (195 μg/100 μL) were prepared and separately adsorbed onto two GG-EDA@pDA sponges (prepared from 200 μL of precursor hydrogel). Both systems were incubated overnight. Subsequently, the samples were frozen at −80 °C and freeze-dried, resulting in GG-EDA@pDA + TMP-G1-[Cys]_6_ and GG-EDA@pDA+TMP-G2-[Cys]_12_ sponges.

### 4.11 Hydrolytic Degradation Study

Hydrolytic degradation study was performed by weighing the freeze-dried samples and then incubating them in DPBS (2 mL, pH 7.4) at 37 °C. At scheduled time intervals (1, 2, 3, 7, and 14 days), each sample was frozen, freeze-dried, and carefully weighed. In parallel, other hydrogels (with and without dendrimers) were exposed, after incubation under the same conditions, to irradiation with NIR light (1.27 W/cm^2^ for 8 min per day). At the end of the programmed period, these samples were also frozen, freeze-dried, and weighed. The degradation of the samples was expressed as a percentage of recovered weight (Wr%), calculated as
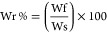


where Wf is the weight of the degraded sample at each time point and Ws is its starting weight. Each experiment was performed in triplicate, and results were expressed as mean value ± standard deviation.

### 4.12 Leach-Out Study

A leach-out study of hydrogels in DPBS (2 mL, pH 7.4) at 37 °C was conducted. For this experiment, 5 μL aliquots were collected at different time intervals (1, 2, 3, 7, 14 days), freeze-dried, and redissolved in DI water (300 μL). Analyses were conducted using a Bruker UltrafleXtreme matrix-assisted laser desorption ionization time-of-flight MALDI-ToF mass spectrometer (Bruker Daltonics, Bremen, Germany) equipped with a SmartbeamII laser (355 nm, UV) in positive mode. Both hydrogels with and without NIR radiation were performed in this study (1.27 W/cm^2^ for 8 min/day). Analyses were conducted using Spherical calibrants (Polymer Factory Sweden AB). Mass spectra were recorded with Flex Control and analyzed with Flex Analysis Version 3.4 (Bruker Daltonics). 2,5-Dihydroxybenzoic acid (DHB) was used as the matrix and prepared by dissolution in tetrahydrofuran (20 mg/mL). Samples were prepared at a ratio of 40:1 of the matrix and analyte. A droplet (5 μL) was deposited on an MPT 284 Target ground steel TF Target plate purchased from Bruker Daltonics.

### 4.13 SEM-EDS of Freeze-Dried Sponges Loaded with Dendrimers

Scanning electron microscopy (SEM) images coupled with energy dispersive X-ray spectroscopy (EDS) were obtained after the samples were frozen in liquid nitrogen, cut with a sharp blade, and freeze-dried.

### 4.14 In Vitro Cytocompatibility Studies

The GG-EDA@pDA, GG-EDA@pDA+TMP-G1-[Cys]_6_, and GG-EDA@pDA+TMP-G2-[Cys]_12_ sponges (derived from 100 mL hydrogel) were placed in a 24-well culture plate and sterilized by UV irradiation at 254 nm for at least 1 h (30 min per side) using a 125 W UV lamp. Then, they were placed in CellCrown well plate inserts (Sigma-Aldrich) and conditioned by treatment with DMEM cell culture medium supplemented with 10% v/v FBS, 1% v/v penicillin-streptomycin solution, 1% v/v glutamine solution, and 0.1% v/v amphotericin B solution for 30 min. Subsequently, the samples were placed in a 24-well plate containing 2.5 × 10^4^ cells (HaCaT and FD-MSCs), seeded 24 h earlier in fresh medium (2 mL). Culture plates were incubated at 37 °C in a humidified incubator with a 5% CO_2_ atmosphere. Cell viability was measured, after 24 and 48 h of culture, by means of the MTS assay following the supplier’s instructions and expressed as a percentage of viability compared to control cells cultured without samples. The live/dead staining protocol was also performed following the supplier’s instructions. Fluorescence images were obtained with an AxioVert200 microscope (Zeiss). Each experiment was performed in triplicate, and results were expressed as mean value ± standard deviation.

### 4.15 In Vitro Hemolysis Test

The hemolysis test was performed by following a procedure already published with some modifications.^57,58^ Briefly, fresh venous blood (400 μL), obtained from a single self-collection performed by the first author, was mixed with DPBS (3 mL, pH 7.4). The mixture was centrifuged at 1200 rpm for 10 min and washed 5 times with DPBS (pH 7.4) until a colorless supernatant was observed. The pellet was recovered with DPBS (900 μL, pH 7.4) to obtain diluted blood for subsequent hemolysis tests. The GG-EDA@pDA sponges were sterilized with UV light (254 nm) and then immersed in DPBS (10 mL, pH 7.4) at 37 °C for 24 h to obtain the hydrogel extract after centrifugation at 1200 rpm for 5 min. The extract was diluted using DPBS (pH 7.4) to obtain different dilution ratios (25%, 50%, and 100%). Diluted blood (20 μL) was added to each extracted sample (1 mL). After incubation at 37 °C for 1 h, the supernatant was collected after centrifugation at 1200 rpm for 10 min. Each collected sample was observed under an optical microscope (AxioVert200 microscope, Zeiss), and the absorbance was measured at 570 nm using a UV–vis spectrophotometer based on the characteristic absorption peak of hemoglobin. DPBS (pH 7.4) and deionized water after incubation with the diluted blood were used as negative and positive controls, respectively. The hemolytic ratio was calculated using the equation:
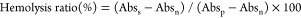
where Abs_s_, Abs_n_, and Abs_p_ are the absorbance of the sample, negative control, and positive control, respectively.

### 4.16 In Vitro Evaluation of Antibacterial Activity

The antimicrobial activity of GG-EDA, GG-EDA@pDA, GG-EDA@pDA+TMP-G1-[Cys]_6_, and GG-EDA@pDA+TMP-G2-[Cys]_12_ against *S. aureus* ATCC33862 and *P. aeruginosa* ATCC25688 was assessed by measuring bacterial growth inhibition in planktonic cultures. A 24-well plate containing BHI (2 mL) and the tested samples (100 mL) were inoculated with approximately 10^5^ colony forming units (CFU)/mL of each sensitive strain. GG-EDA@pDA, GG-EDA@pDA+TMP-G1-[Cys]_6_, and GG-EDA@pDA+TMP-G2-[Cys]_12_ hydrogels were irradiated with NIR light (1.27 W/cm,^2^ 8 min) for a single treatment or for three treatment cycles (one irradiation every two hours). Following NIR treatment, the plates inoculated with *S. aureus* were incubated at 37 °C for 24 h, while the plates inoculated with *P. aeruginosa* were incubated at 25 °C for 24 h. The unirradiated samples of GG-EDA@pDA, GG-EDA@pDA+TMP-G1-[Cys]_6_, and GG-EDA@pDA+TMP-G2-[Cys]_12_ were inoculated and incubated under the same conditions. BHI alone was used as a negative control, while BHI was inoculated with each sensitive strain as a positive control. After incubation, 1 mL from each well was subjected to serial decimal dilution (1:10) in Ringer’s solution (Oxoid). Cell suspensions were plated on Baird Parker (BP) added with rabbit plasma fibrinogen (RPF) to enumerate *S. aureus*, and on *Pseudomonas* Agar Base (PAB) added with Cetrimide Fucidin Cephaloridine (CFC) supplement, for *P. aeruginosa*. The experiments were conducted in triplicate and were repeated twice.

### 4.17 Statistical Analysis

At least three independent replications for each data set of all experiments were performed, and results are reported as mean ± Standard Deviation. The cytocompatibility test and the microbial count data were subjected to analysis of variance (ANOVA) and compared pairwise using Tukey’s test. The level of statistical significance was set at *p* ≤ 0.001. The software used for statistical data processing was XLStat software, version 2019.2.2 (Addinsoft, New York, USA).

## Supporting Information Available

Additional experimental figures including SEM, oscillation amplitude and frequency sweep analysis at 25 °C, temperature ramp analysis, spectra resulting from the radical scavenging assay with ABTS^•+^, ATR-FTIR spectra, live/dead staining assay of HaCaT and FD-MSC cells, ^1^H NMR spectrum, and Table S1 with DDEDA mol % values obtained with ^1^H NMR analysis and TNBS assay (PDF)
