# Supplementary material for: Antimicrobial NIR-Responsive Hydrogels Based on Gellan Gum and Bis-MPA Polyester Dendrimers
Source: ACS Appl Mater Interfaces. 2025 Apr 7;17(15):22448–63. doi: 10.1021/acsami.5c02386 (PMC12012787; doi:10.1021/acsami.5c02386)
Supplement: Supplementary file 1 — am5c02386_si_001.pdf [file am5c02386_si_001.pdf]

Supporting Information

Antimicrobial NIR-Responsive hydrogels based on Gellan Gum and bis-MPA polyester dendrimers

Giuseppina Biscari[a], Natalia Sanz del Olmo[b], Fabio S. Palumbo[a], Raimondo Gaglio[c], Giuliana Garofalo[c], Giovanna Pitarresi[a], Calogero Fiorica[a] and Michael Malkoch*[b]*

[a]Department of Biological Chemical and Pharmaceutical Science and Technology (STEBICEF), University of Palermo, Via Archirafi 30-32, Palermo, 90123, Italy

[b]School of Engineering Sciences in Chemistry, Biotechnology and Health (CBH), Department of Fibre and Polymer Technology, Division of Coating Technology, KTH Royal Institute of Technology, Teknikringen 56, Stockholm, SE-100 44 Sweden

[c]Department of Agricultural, Food and Forest Sciences (SAAF), Università degli Studi di Palermo, Viale delle Scienze, Palermo, 90128 Italy

* Corresponding authors. E-mail: calogero.fiorica@unipa.it (Calogero Fiorica); malkoch@kth.se (Michael Malkoch).

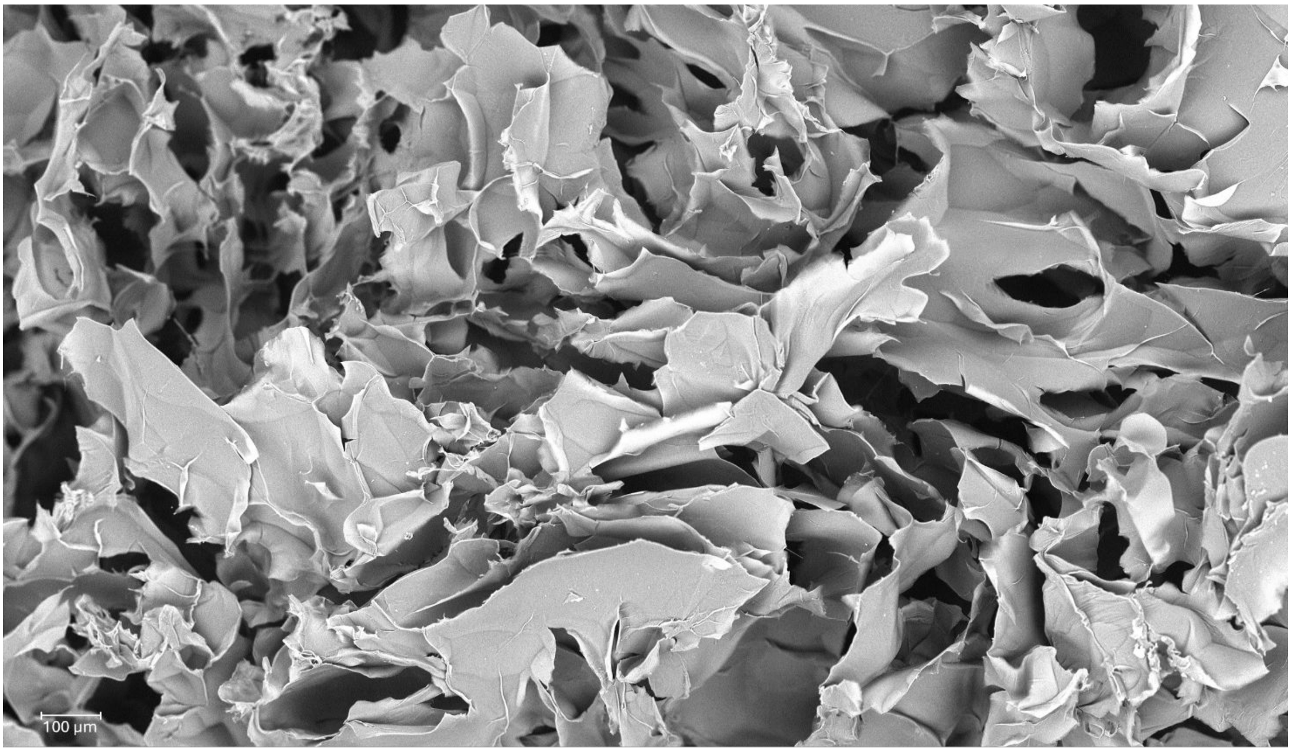

Figure S1. SEM analysis of GG-EDA@pDA freeze-dried sponge

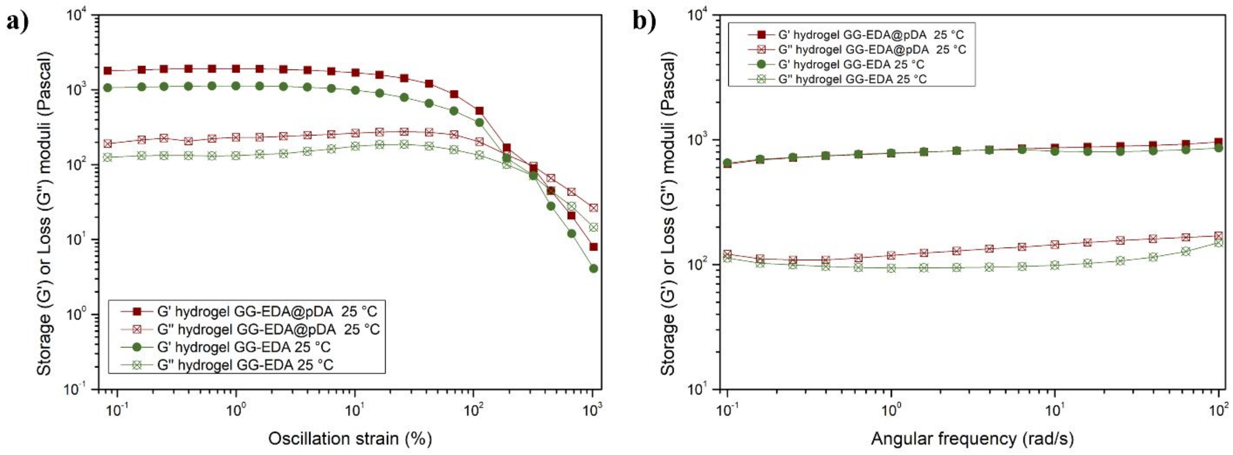

Figure S2. Oscillation amplitude (**a**) and frequency sweep (**b**) analysis at 25 °C of GG-EDA and GG-EDA@pDA

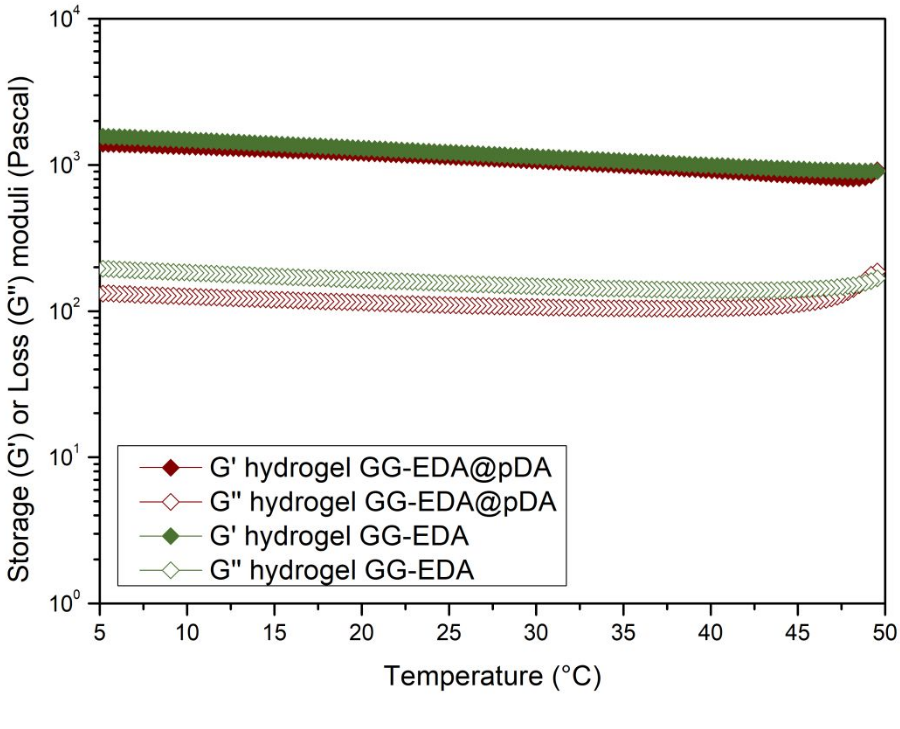

Figure S3. Temperature ramp analysis of GG-EDA and GG-EDA@pDA

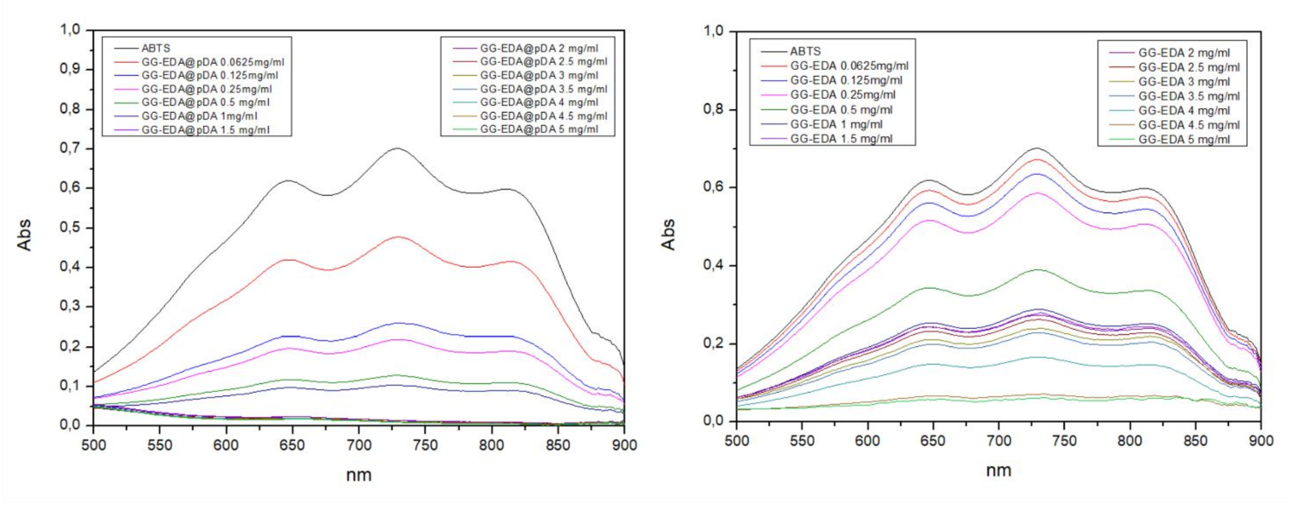

Figure S4. Spectra resulting from the radical scavenging assay with *ABTS*$^{\bullet+}$ performed on GG-EDA and GG-EDA@pDA

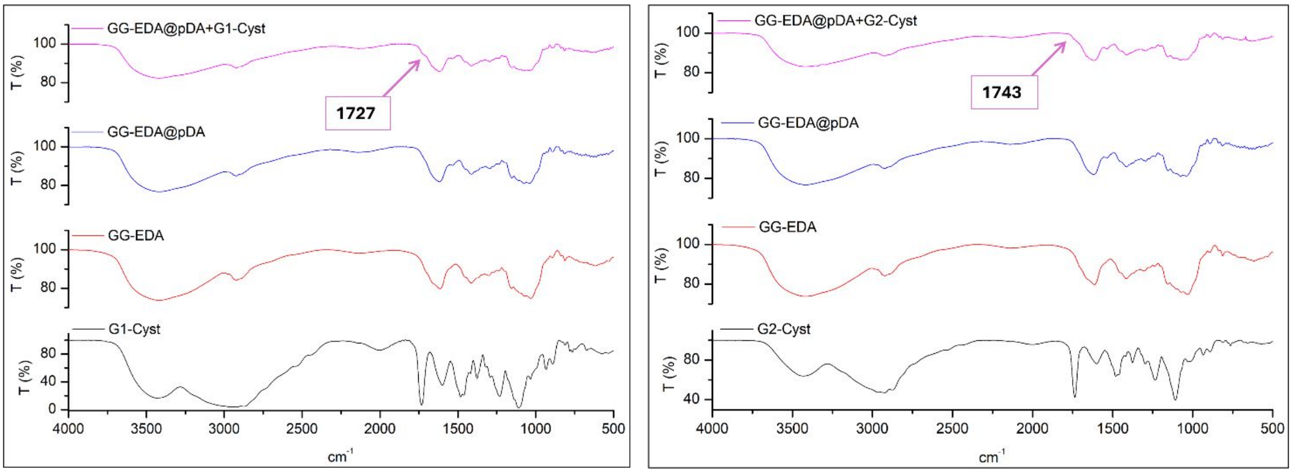

Figure S5. ATR-FTIR spectra of GG-EDA, GG-EDA@pDA, TMP-G1-[Cys]$_6$, TMP-G2-[Cys]$_{12}$, GG-EDA@pDA+TMP-G1-[Cys]$_6$, and GG-EDA@pDA+TMP-G2-[Cys]$_{12}$

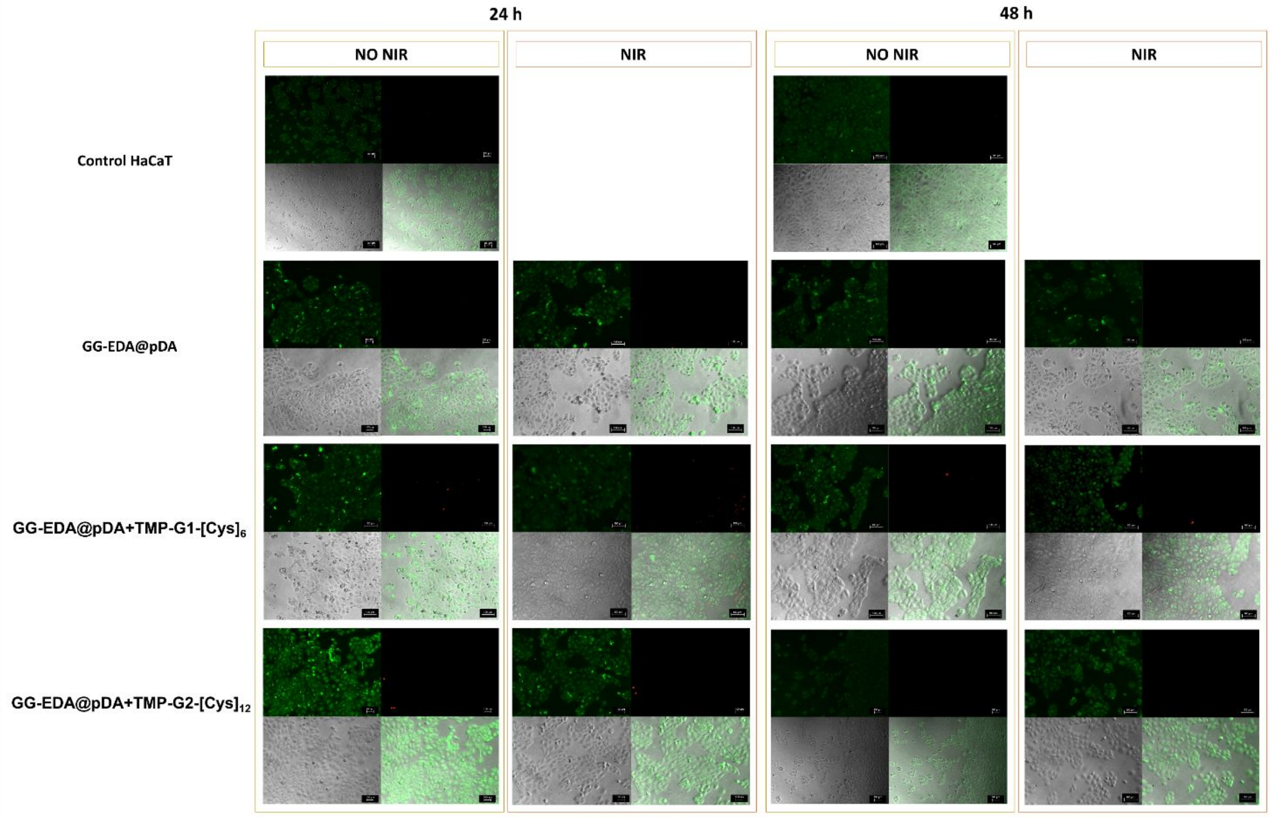

Figure S6. Live/Dead staining assay of HaCaT cells after 24 and 48 hours of co-incubation with GG-EDA@pDA, GG-EDA@pDA+TMP-G1-$[Cys]_6$, GG-EDA@pDA+TMP-G2-$[Cys]_{12}$ after and without NIR treatment

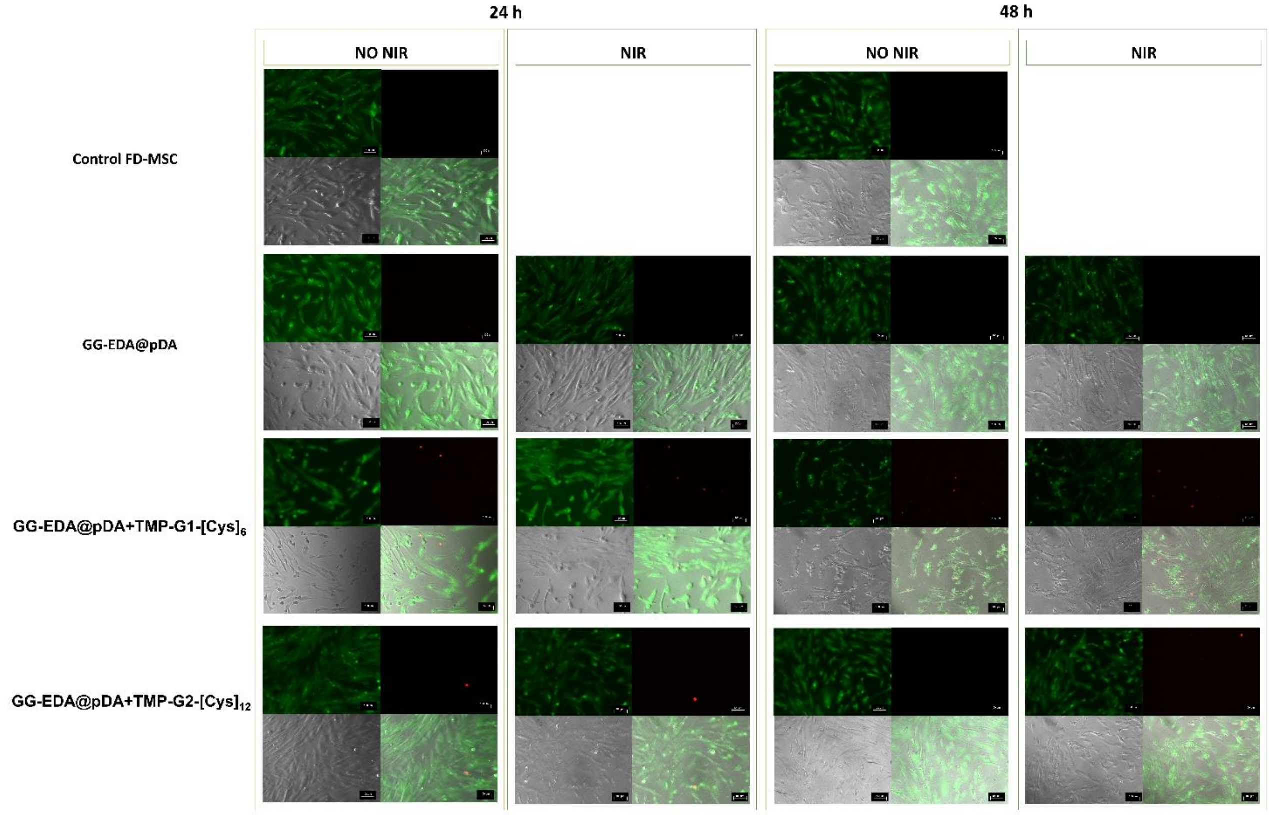

Figure S7. Live/Dead staining assay of FD-MSC cells after 24 and 48 hours of co-incubation with xerogels GG-EDA@pDA, GG-EDA@pDA+TMP-G1-[Cys]$_6$, GG-EDA@pDA+TMP-G2-[Cys]$_{12}$ after and without NIR treatment

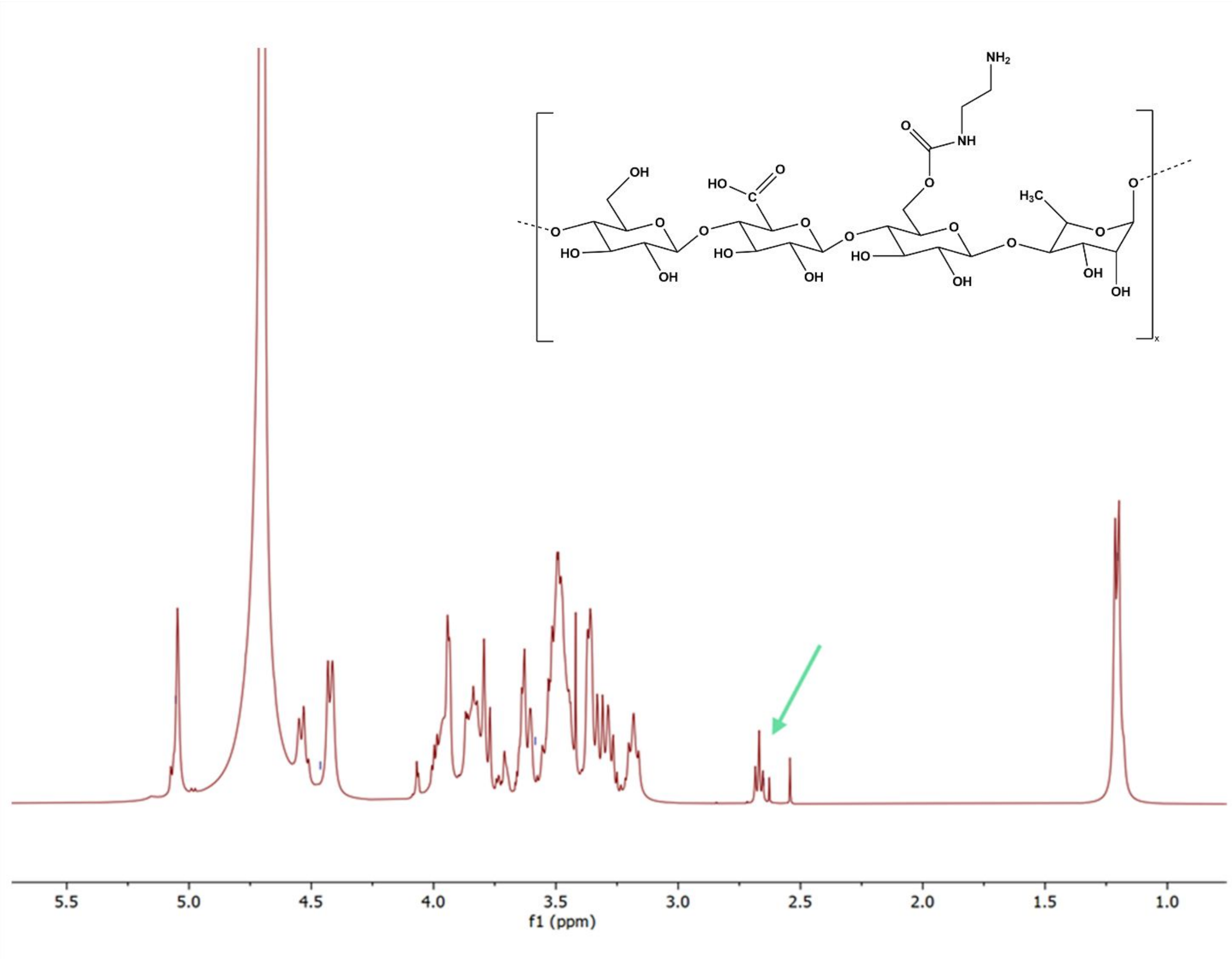

Figure S8. ^{1}H-NMR spectrum of GG-EDA

Table S1. DD$_{EDA}$ mol% values obtained with ^{1}H-NMR analysis and TNBS assay

**Method**	**DD$_{EDA}$ mol% value**	**Standard deviation**
**^{1}H-NMR**	28	±4
**TNBS**	29	±2

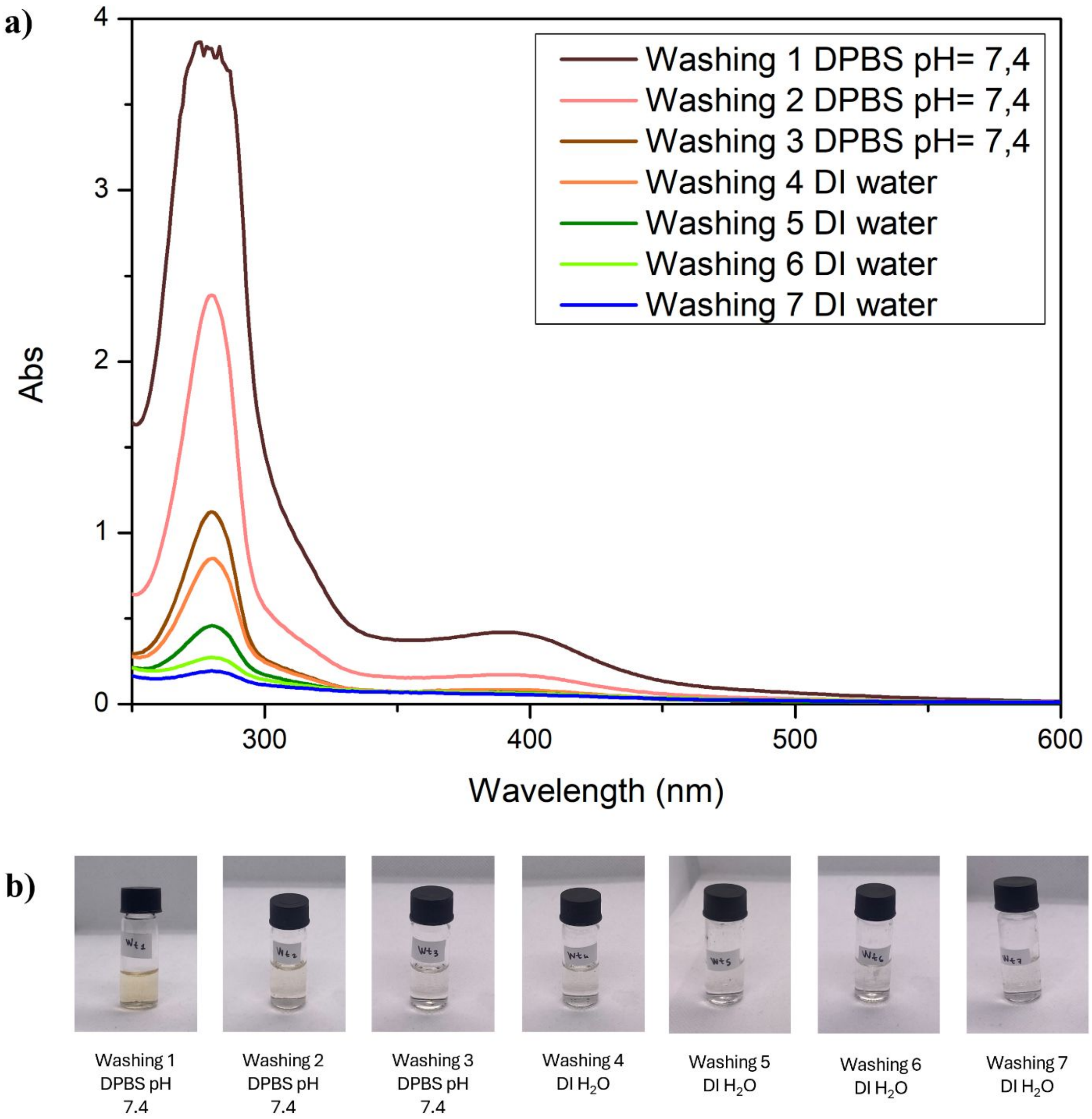

Figure S9. UV-vis spectra to determine the decreasing presence of excess dopamine in the wash waters (**a**); Photos of the wash waters (**b**)
